# Tough places and safe spaces: Can refuges save salmon from a warming climate?

**DOI:** 10.1002/ecs2.4265

**Published:** 2022-11-09

**Authors:** Marcía N. Snyder, Nathan H. Schumaker, Jason B. Dunham, Joseph L. Ebersole, Matthew L. Keefer, Jonathan Halama, Randy L. Comeleo, Peter Leinenbach, Allen Brookes, Ben Cope, Jennifer Wu, John Palmer

**Affiliations:** 1 US Environmental Protection Agency, Pacific Ecological Systems Division, Corvallis, Oregon, USA; 2 US Geological Survey, Forest and Rangeland Ecosystem Science Center, Corvallis, Oregon, USA; 3 University of Idaho, Department of Fish and Wildlife Sciences, College of Natural Resources, Moscow, Idaho, USA; 4 Oak Ridge Institute for Science and Education/US Environmental Protection Agency, Pacific Ecological Systems Division, Corvallis, Oregon, USA; 5 US Environmental Protection Agency, Seattle, Washington, USA

**Keywords:** climate, cold-water, HexSim, individual-based model, migration, refuge, salmon, trout

## Abstract

The importance of thermal refuges in a rapidly warming world is particularly evident for migratory species, where individuals encounter a wide range of conditions throughout their lives. In this study, we used a spatially explicit, individual-based simulation model to evaluate the buffering potential of cold-water thermal refuges for anadromous salmon and trout (*Oncorhynchus* spp.) migrating upstream through a warm river corridor that can expose individuals to physiologically stressful temperatures. We considered upstream migration in relation to migratory phenotypes that were defined in terms of migration timing, spawn timing, swim speed, and use of cold-water thermal refuges. Individuals with different migratory phenotypes migrated upstream through riverine corridors with variable availability of cold-water thermal refuges and mainstem temperatures. Use of cold-water refuges (CWRs) decreased accumulated sublethal exposures to physiologically stressful temperatures when measured in degree-days above 20, 21, and 22°C. The availability of CWRs was an order of magnitude more effective in lowering accumulated sublethal exposures under current and future mainstem temperatures for summer steelhead than fall Chinook Salmon. We considered two emergent model outcomes, survival and percent of available energy used, in relation to thermal heterogeneity and migratory phenotype. Mean percent energy loss attributed to future warmer mainstem temperatures was at least two times larger than the difference in energy used in simulations without CWRs for steelhead and salmon. We also found that loss of CWRs reduced the diversity of energy-conserving migratory phenotypes when we examined the variability in entry timing and travel time outside of CWRs in relation to energy loss. Energy-conserving phenotypic space contracted by 7%–23% when CWRs were unavailable under the current thermal regime. Our simulations suggest that, while CWRs do not entirely mitigate for stressful thermal exposures in mainstem rivers, these features are important for maintaining a diversity of migration phenotypes. Our study suggests that the maintenance of diverse portfolios of migratory phenotypes and cool- and cold-water refuges might be added to the suite of policies and management actions presently being deployed to improve the likelihood of Pacific salmonid persistence into a future characterized by climate change.

## INTRODUCTION

The Earth is warming rapidly (IPCC, 2019) and many species are being forced to acclimatize, adapt, move, or die in response (Beever et al., 2017; Kearney & Porter, 2009; Wiens, 2016). As species experience greater exposure to climate change, increasing attention is being paid to the role of thermal refuges and climate refugia (Morelli et al., 2016; Reside et al., 2019). Refuges offer respite from acute and chronic exposures to stressful and potentially lethal conditions, but the availability of refuges does not guarantee a benefit will be accrued (Beever et al., 2017; Kearney & Porter, 2009; Sears et al., 2016; Wiens, 2016). For example, the ability of a species to exploit a refuge depends on movement constraints imposed by the external environment, individual cognitive and sensory abilities, and internal drivers of movement such as energy gain, survival, and reproduction (Fey et al., 2019; Nathan et al., 2008). The potential for refuges to enhance population viability is of great interest (Berryman et al., 2006; Davis et al., 2013; Sears et al., 2016). However, an appreciation of the potential for refuges to improve outcomes for species responding to climate change, particularly those that migrate, will require consideration of complex interacting processes such as spatially and temporally variable migration route conditions and individual behaviors. These types of interactions are not easily elucidated using conventional observational or experimental approaches.

Variability in migratory behavior of individuals such as refuge use or stopover frequency (i.e., migratory phenotypes) can lead to differing survival and fitness outcomes. Various aspects of migratory phenotypes have been described and quantified. For example, migratory phenotypes in long-distance reproduction migrations of birds have been defined by route selection, temporal variation in departure and arrival, age at onset, stopover decisions, and flight speeds (Lindström et al., 2016; Shamoun-Baranes et al., 2010). Consequences of behavioral decisions during migration can include variable exposure to predation risk (Sabal et al., 2021), spatial and temporal overlap with key resources and/or stressors (Rivrud et al., 2018), and, particularly for ectotherms in thermally diverse migratory corridors, energy expenditure (Crozier et al., 2017). These factors can collectively influence survival and reproductive potential. Here, we apply the concept of migratory phenotypes to an aquatic system with the goal of describing the collection of individual migratory behaviors that influence an individual’s exposure to stressful temperatures and rates of energy consumption.

Stream temperatures in the Pacific Northwest, USA, are warming (Isaak et al., 2012) and are strongly linked to increasing vulnerability of Pacific salmonids (*Oncorhynchus* spp.) to climate change (Crozier et al., 2019; Isaak et al., 2016). Rates of water temperature warming in the region are highest in the summer (0.22°C per decade) and are strongly linked to air temperatures and river discharge (Isaak et al., 2012). Cold-water refuges (CWRs) have been suggested as an increasingly critical resource for cold-water fishes, including salmonids, as their exposure to warming temperatures increases (Ebersole et al., 2020; Isaak et al., 2016). In fact, upstream-migrating salmonids are frequently observed using CWRs when river temperatures increase to physiologically stressful levels (Berman & Quinn, 1991; Frechette et al., 2018; Goniea et al., 2006; Keefer et al., 2009, 2018; Torgersen et al., 1999). Yet in other cases, upstream-migrating salmonids are observed to bypass available thermal refuges when river temperatures are high (Goniea et al., 2006; Keefer et al., 2009).

**FIGURE 1:**
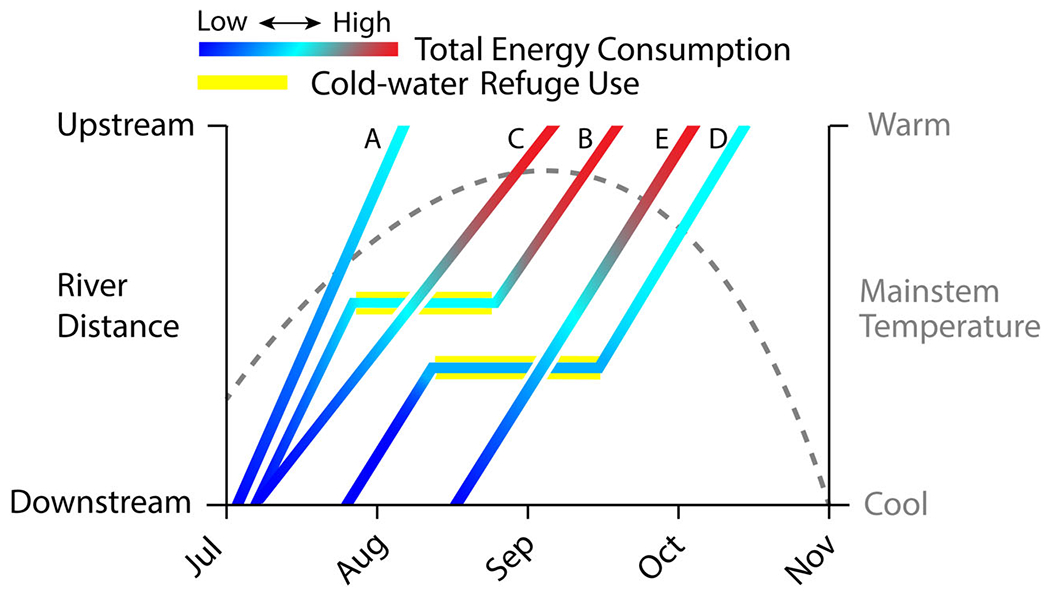
Each line represents an example of a hypothetical migratory phenotype (migration strategy) of upstream migration within the modeled system with color representing total energy used at that river distance and date. Distance is represented as a fish’s position in the Columbia River between Bonneville Dam (downstream) and the Snake River confluence (upstream). Migration timing is indexed by month. The dashed gray line corresponds to the temperature of the Columbia River. Energy-conserving strategies end in cyan and energy non-conserving strategies end in red. Arrival date at the upstream terminus is an emergent outcome of migration strategy. Migratory phenotypes can vary by swim speed, migration timing in relation to temperature of the migration corridor, and cold-water refuge use (yellow highlights).

In this study, we employ a spatially explicit, individual-based model (IBM) (Snyder et al., 2019) to investigate the potential for thermal refuges to benefit upstream-migrating Pacific salmonids in the Pacific Northwest, USA. We simulated behavioral thermoregulation by summer steelhead (*Oncorhynchus mykiss*) and fall Chinook Salmon (*Oncorhynchus tshawytscha*) migrating through the lower Columbia River. Fall Chinook Salmon swim through the lower Columbia River migration corridor from August to October and spawn from October to December (Jepson et al., 2010). Summer steelhead move upstream from June to October, and although they have a protracted migration and are spring spawners, most migrate through the Columbia River by November (Robards & Quinn, 2002). We evaluated the consequences of thermal refuge use by tracking the thermal exposures of individual fish along migratory pathways. Thermal exposure was thus an outcome of how individual fish among various upstream migratory phenotypes experienced mainstem river temperatures and cold-water refuge temperatures in space and time (Figure 1). In this study, we define migratory phenotypes by behaviors including timing of upstream migration, migration duration, and patterns of cold-water refuge use. Our simple conceptual framework allows exploration of how refuge use can be a cost or a benefit, depending on riverscape conditions (Fausch et al., 2002) and migratory phenotypes determined by individual fish behaviors (Figure 1).

As we model them, migratory phenotypes will vary along a continuum (e.g., slow to fast swim speed). However, for illustrative purposes, we identify five distinct migratory phenotypes corresponding to specific physiological attributes and behaviors. These include phenotype A (early entry timing, fast swim speed, and no cold-water refuge use), phenotype B (early entry timing, fast swim speed, and cold-water refuge use), phenotype C (early entry, slow swim speed, and no cold-water refuge use), phenotype D (intermediate entry timing, fast swim speed, and cold-water refuge use), and phenotype E (later entry timing, fast swim speed, and no cold-water refuge use). Comparisons between phenotypes A and B will illustrate how cold-water refuge stopover might influence the total energy used for an individual entering the Columbia River early in the migration. Comparing phenotypes A and C will illustrate how swim speed or time spent migrating could lead to higher total energy expenditure. Contrasting results from phenotypes A and E will illustrate how entry timing could influence total energy consumption, even if swim speed and cold-water refuge use are identical. Comparing phenotypes B and D will illustrate how entry timing and cold-water refuge use could interact to be the most beneficial to total energy consumption. To address the variation in the magnitude and spatial heterogeneity of thermal conditions, we considered current and future warming scenarios, and systems with and without CWRs. By simulating movement behavior and spatiotemporal heterogeneity of thermal exposures within a riverscape (henceforth thermalscape), we were able to evaluate how changing conditions influence (1) the diversity of energy-conserving migratory phenotypes in two species of upstream-migrating salmonids, (2) acute and accumulated exposures to very warm temperatures, and (3) net energy loss and reserves available for reproduction. Based on previous studies of migrating summer steelhead and fall Chinook Salmon that demonstrated behavioral differences in timing and thermal refuge use (Keefer et al., 2018), we hypothesized that our model would support the existence of a diversity of energy-conserving migratory phenotypes. We also hypothesized that CWRs reduce the proportion of a population with higher accumulated exposure and reduce mortality from acutely high temperatures. Given the differences in spawn timing (fall-run Chinook Salmon need to arrive at their spawning grounds much earlier than spring-spawning summer steelhead), we expected that refuge benefits would vary between species. Lastly, we anticipated that cold-water refuge use would be energetically beneficial because populations migrating through thermalscapes with CWRs should consume less energy than those for whom CWRs are unavailable.

## MATERIALS AND METHODS

### Study site

The Columbia River basin is inhabited by numerous populations of anadromous salmon and steelhead listed as endangered or threatened under the US Endangered Species Act (NMFS, 2016), and that have been the focus of extensive research and conservation efforts. These culturally, economically, and ecologically important species (Bottom et al., 2009; Stouder et al., 1997; Ween & Colombi, 2013) are increasingly likely to encounter warm temperatures at sublethal levels during summer and fall upstream reproductive migrations (Isaak et al., 2012). Average summer river temperatures have increased by ~2.5°C since the 1950s (Isaak et al., 2016; Keefer et al., 2018; Quinn & Adams, 1996).

**FIGURE 2:**
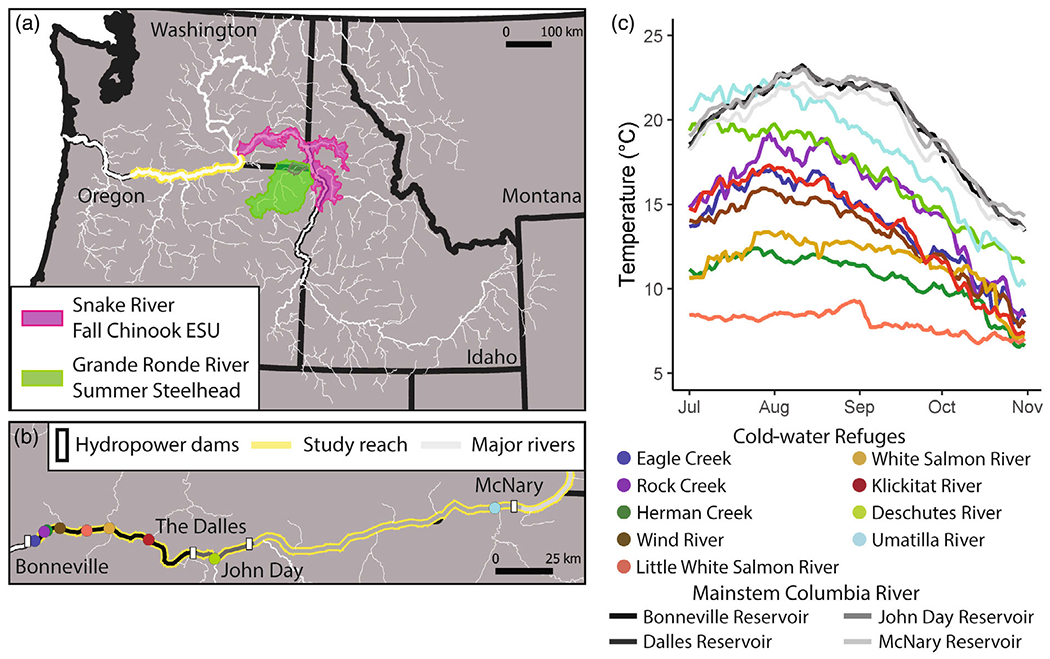
Study reach shown within the context of the mainstem Columbia River and major tributaries (a). Pink and green polygons highlight available spawning area for Grande Ronde River summer steelhead and Snake River fall Chinook Salmon evolutionarily significant units (ESUs). Colored points in (b) correspond to significant cold-water refuges (CWRs) between Bonneville Dam and the Snake River confluence. (c) The mean daily temperature for modeled portion of mainstem Columbia River reservoirs (gray-scale lines) and CWRs (colored lines corresponding to points in [b]).

Much of the Columbia River migration corridor has been modified by a series of hydropower dams and their associated reservoirs (Figure 2). We parameterized the model to run starting at Bonneville Dam, located 235 river kilometers (rkm) from the Pacific Ocean and ending at the Snake River confluence (537 rkm). We selected the lower Columbia reach for our study because a HexSim model had already been developed for this system (Snyder et al., 2019) and because the Snake River confluence is important ecologically, and from the standpoint of salmon management (NMFS, 2016). Along this 288-rkm section of the lower Columbia River, the primary CWRs are formed where cooler tributaries enter the mainstem (Keefer et al., 2018; Snyder et al., 2019). This class of CWRs consists of tributary and plume components, which lay adjacent to (tributaries) and within (plumes) the Columbia River itself. The reservoirs’ reaches are generally well mixed vertically (Yearsley, 2009), although there is episodic thermal stratification near John Day and McNary Dams (US Army Corps of Engineers, unpublished data) and likely do not provide CWRs for migrating salmonids in deep sections (Keefer et al., 2019). Low diurnal variability (<0.5°C on average) means that little opportunity exists for nighttime escape from warm temperatures (University of Washington, 2018).

For this study, we identified CWRs that met accessibility and temperature criteria defined by the US Environmental Protection Agency (2020). CWRs in the model had mean August discharge >0.28 m^3^/s (~10 cubic feet per second) estimated as likely to provide adequate depths and volumes for thermoregulation of adult salmon and steelhead for tributary and plume components (Figure 2, Table 1). Mean August discharge of tributaries comprising CWRs were approximated using US Geological Survey (USGS) gage data, USGS StreamStats, or from NHDPlus depending on availability (USGS, 2016, 2018). Cold-water refuge volumes were estimated using both field surveys of depth and temperature and plume/tributary models that incorporated tributary discharge and confluence morphology (see US Environmental Protection Agency, 2020, for additional details). In the models, estimated cold-water refuge volumes were unchanging through time. For consistency and applicability to regional water temperature guidance, we defined a cold-water refuge as a location that had a temperature threshold of 2°C less than that of the Columbia River at some time during the adult salmon and steelhead migration. We identified nine CWRs, which were accessible to migrating adult salmonids and met the temperature criteria (Table 1, Figure 2). Seven of these occur in the Bonneville Reservoir, one is in the Dalles Reservoir, and one is in the John Day Reservoir. Between July and November, mean temperature values for these CWRs ranged from 8.0 to 18.4°C. Mean Columbia River temperatures measured at Bonneville Dam from 1996 to 2015 ranged from 16 to 23°C (Keefer et al., 2018).

### Model simulation

We ran simulations using an updated version of the spatially explicit individual-based migration corridor simulation model described in the study by Snyder et al. (2019). This simulation model incorporates key elements of thermal niche modeling (Kearney et al., 2010; Kearney & Porter, 2009) and movement ecology (Nathan et al., 2008) within a two-dimensional thermalscape exhibiting complex spatial and temporal thermal dynamics. Modifications made to that model for this investigation include (1) expanding the study area from the Bonneville Reservoir reach (73 rkm long) to the lower Columbia River from Bonneville Dam to the Snake River confluence (288 rkm long); (2) incorporation of energetic costs associated with fish passage through fishways at hydropower dams; and (3) limited re-parameterization of the model’s entry timing and body mass parameters to the specific salmonid populations of interest.

The migration corridor simulation model is a hybrid mechanistic–probabilistic model designed to account for many dynamic and interacting processes. The model was developed in the HexSim development environment (Schumaker & Brookes, 2018). Simulated individuals may be assigned any number of traits that can change through time, for example, as a result of interactions with the environment or with other organisms. Traits include parameters such as swim speed, body size, and river reach entry date. Full accounting and description of traits and model parameter details are provided in Snyder et al. (2019). The model was mechanistic to the extent possible given limitations in available data and confines of knowledge regarding the likelihood of an individual’s behavior within certain environmental states. When insights were insufficient for the construction of mechanistic model components, we substituted probability-based decision tress. Probabilistic mechanisms were based on distributions of values estimated from observed data. Examples of probabilistic model components included the likelihood of behavioral thermoregulation, individual residence times in a cold-water refuge, and the time spent swimming through dam tailraces and fishways (see Snyder et al., 2019, Appendix S1 and S2). An important attribute of the model is that it can integrate multiple complex and interacting drivers (e.g., thermal exposure, migration phenology, dam passage timing, and propensity to thermoregulate) experienced at the scale of individuals, all within a detailed spatially nuanced environment. And the model’s design makes it suitable for generating defensible forecasts of population-scale responses to novel future conditions.

We used the model to simulate a single stage of the salmonid life cycle, adult upstream migration, through the lower Columbia River corridor to the Snake River confluence. The lower Columbia River corridor is used extensively by many threatened and endangered salmonids from the Snake and upper Columbia River. Species- and population-specific migration corridor entry timing and initial body masses were assigned based on empirical data (Jepson et al., 2010; Keefer et al., 2009). Once individuals entered the simulated migration corridor, simulated fish moved between the river mainstem and available CWRs depending on their history of thermal exposure, propensity for behavioral thermoregulation, and species-specific thermal tolerances (Goniea et al., 2006; Keefer et al., 2009). During each time step, a probability-based decision table was used to determine the individual fish movement behavior (e.g., move upstream, move to cold-water refuge, and leave cold-water refuge). Initial parameterization of an individual’s movement behavior was based on reference empirical data from adult steelhead and Chinook Salmon obtained using intragastric radio data storage transmitters (Goniea et al., 2006; Keefer et al., 2018; Snyder et al., 2019). Refuge use was also influenced by the location of the fish relative to the refuge and the current fish density in a refuge (number of fish per volume of cold water). Simulated fish were assigned target arrival times at spawning grounds based on spawn-timing records for these species (Groves & Chandler, 1999; Keefer et al., 2018). These target arrival times were used in the model to increase a simulated individual’s motivation to travel upstream as the target arrival time got closer. While actively migrating, simulated fish consumed energy at a rate corresponding to their mass and thermal exposure, with the latter in turn affecting an individual’s propensity to seek and travel toward available CWRs. Energy consumption rates were lower while fish resided in CWRs because metabolic costs are minimized near optimum temperatures and from lower activity costs (Deslauriers et al., 2017; Plumb, 2018; Stewart & Ibarra, 1991).

**FIGURE 3:**
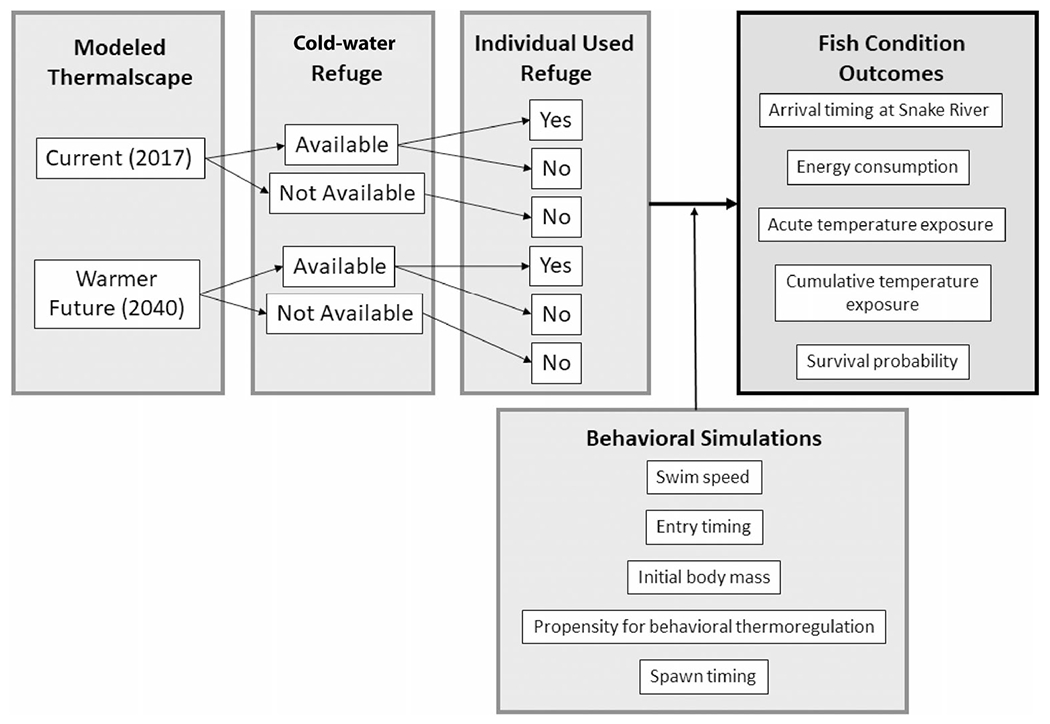
A simplified version of the experimental setup. Each box represents a factor that, when combined with the other factors, can influence the modeled condition outcomes.

The spatially dynamic IBM was developed and parameterized with empirical data, and was comprised of the Wisconsin Bioenergetics framework (Deslauriers et al., 2017; Plumb, 2018; Stewart & Ibarra, 1991) and a simplified fish behavior sub-model (Snyder et al., 2019). Together, these model components made it possible to estimate the effects of behavioral thermoregulation and warmer mainstem Columbia River temperatures on fish condition outcomes. Metrics the model used to record condition outcomes include percent energy used, acute and accumulated thermal exposure, and survival (Figure 3).

We modified simulated fish populations initially parameterized to represent interior Columbia River populations named for the timing of their upstream migration: fall Chinook Salmon (Jepson et al., 2010) and summer-run steelhead (Snyder et al., 2019). These changes were intended to better capture the life history details of two specific stocks: Grande Ronde River summer steelhead and Snake River fall Chinook Salmon. These stocks frequently use CWRs because their adult migration timing coincides with warmer Columbia River temperatures, which often reach 19–22°C during summer, a range linked with higher stress and modified behavior (McCullough et al., 2009; Richter & Kolmes, 2005). To simulate Grande Ronde River summer steelhead, we modified the existing summer steelhead population parameters (which previously represented all Columbia River summer steelhead populations): entry weights and timing were 5092 ± 1674 g (mean ± SE) and August 15 ± 15 days, respectively. To simulate Snake River fall Chinook Salmon, we modified the existing Chinook Salmon population entry parameters (which previously represented all Columbia River fall Chinook Salmon populations) to 4279 ± 2088 g (weight) and September 3 ± 6.5 days (timing). Fork lengths from radio-tagged fish of known origin (Jepson et al., 2010; Keefer et al., 2009) were converted to total length using published equations (Conrad & Gutmann, 1996; Mahmoudi et al., 2014). The total length was then used to estimate the distribution of initial body masses (Schneider, 2000; Seelbach, 1993). Species-specific differences in propensity for behavioral thermoregulation, swim speed, and spawn timing were unchanged from the more general models. Within CWRs, high densities of individuals can aggregate and potentially limit behavioral thermoregulation by later-arriving individuals. Previous simulations (Snyder et al., 2019) suggested that fish were not limited by the total volume of CWRs available and therefore condition outcomes were unlikely to be influenced by density-dependent effects from co-occurring populations not represented in our simulations.

Migrating adult salmonids in the Columbia River can spend a significant portion of their upstream journey passing through fishways at hydropower structures (Connor et al., 2019; Crozier et al., 2017; Keefer et al., 2004; Keefer et al., 2021). Between Bonneville Dam and the Snake River confluence, fish migrate past three hydropower dams (the Dalles, John Day, and McNary). Passage through dam tailraces and fishways decreases migration speed and increases energetic costs (Brown et al., 2006). Despite tailraces comprising a small portion of a reservoir’s length, they can consume a disproportionate quantity of fish energy and time because of their increased hydrologic complexity and dynamic flows. Hydropower passage time was modeled as the sum of the time spent in the tailrace and fishways at each dam. Increased energetic costs at the dams were incorporated by multiplying the energy consumed per hour by scaling factors specific to the tailrace (1.62) and fishway (1.26) (Keefer et al., 2017). Fish behavior in tailraces was categorized by arrival time at the tailrace (day or night) and by overall passage speed (fast, slow, and very slow) (Crozier et al., 2017). Each of the six resulting combinations of arrival time and passage speed were parameterized from different distributions characterized by a mean and SD (see Appendix S2). We iteratively adjusted fishway passage timing so that overall tailrace and dam passage times agreed with observed median passage times for radio-tagged adult salmon and steelhead (19 h per dam) (±15%) summarized in Keefer et al. (2021).

The migration corridor simulation model was re-tuned following Snyder et al. (2019) due to the larger spatial extent and incorporation of hydropower dams. Model tuning was performed for average recent temperatures and for the upriver interior Columbia River basin fall Chinook Salmon and summer steelhead populations rather than specific stocks or years. Model tuning largely followed the same steps as in Snyder et al. (2019), which consisted of modifying tuning parameters based on their effect on emergent model outcomes compared with empirical observations. For more details, see Appendix S2.

### Thermalscapes

We simulated modified thermalscapes to further our understanding of how cold-water refuge availability influenced fish condition outcomes during upstream migration under current and predicted future temperatures in the lower Columbia River. To examine the capacity for CWRs to influence present-day fish condition, we compared model outcomes from a simulation of the current thermalscape to one with no available CWRs. We also simulated a future Columbia River thermalscape with and without refuges in order to better quantify the capacity of CWRs to mitigate for warmer predicted Columbia River temperatures. The current Columbia River thermalscape was developed with water temperature data from the year 2017 because it was one of the warmer years in the recent past (Appendix S1). The model used a continuous hourly temperature time series for the Columbia River and cold-water refuge tributaries and cold-water refuge tributary plumes. Temperature observations from US Army Corps of Engineers water quality monitoring stations at dam forebays were downloaded from DART (http://www.cbr/washington.edu/dart) and used to model temperatures in the four lower Columbia River reservoirs. For current thermalscapes, cold-water refuge tributary temperatures were estimated using NorWest measured temperatures (Isaak et al., 2017) and extrapolated to cold-water refuge plumes via a simple mixing model that included the mainstem Columbia River temperature. The future Columbia River thermalscape was based on predictions for the year 2040, using an estimate of 1°C increase from present-day temperatures (Payne et al., 2004). This estimate did not account for potential spatial variability in warming (Steel et al., 2019). See Appendix S1 for more details on estimation methods for creation of the thermalscapes.

### Migratory phenotypes

Migratory phenotypes can incorporate multiple attributes, including a range of entry times, spawn timing, swim speeds, body sizes, and behavioral thermoregulation traits. These phenotypes thus exist within an *n*-dimensional attribute space. Here we consider a subset of the potential migratory behaviors or traits compromising a migratory phenotype, by focusing on attributes relevant to the temperature-tolerant phenotypes concept introduced in the study by Keefer et al. (2018). We evaluated the influence of CWRs on the diversity of migratory phenotypes by evaluating two emergent model outcomes: (1) phenotypic space of the proportion of fish successful at energy conservation and (2) the range of arrival dates at the Columbia and Snake River confluence (our model terminus). For successful energy-conserving individuals, the change in area of phenotypic space defined by entry timing relative to the maximum temperature of the Columbia River and travel time outside of CWRs was used to measure the diversity of migratory phenotypes. We labeled individuals as successful at energy conservation when they consumed less than the 25th percentile of energy use observed for the simulated population under current conditions with CWRs. These energy-conserving phenotypes exhibited <25% energy loss (steelhead) and <17% energy loss (Chinook Salmon). Our use of distribution-based energy conservation thresholds reflected the lack of empirical evidence necessary to unequivocally link available energy to successful spawning at the spawning grounds. However, existing studies of salmonid spawning and pre-spawning mortality in the Columbia River basin indicate that significant quantities of energy are expended during migration and pre-spawn holding upstream of the Snake River–Columbia River confluence, and that successful spawners are likely to be those with high energy densities (e.g., Bowerman et al., 2017; Connor et al., 2019).

### Acute and accumulated exposure

We evaluated accumulated exposure (cumulative degree-days) to warm but sublethal temperatures across a range of implied thermal tolerance thresholds of 20, 21, and 22°C. Cumulative degree-days were calculated as the sum of the hourly temperatures from a modeled individual’s temperature exposure time series, when temperature was above the thresholds (e.g., 22°C). Hourly sums were converted to days by dividing by 24. Acute temperature stress can directly impact an individual’s probability of surviving (Jager, 2011; Railsback et al., 2009; Sullivan et al., 2000). An individual can experience a higher risk of mortality, imposed here as a probability of survival estimated from lab studies, when exposure to high temperatures persisted for 24 h or more (Railsback et al., 2009). In the model, survival is a probability based on the relationship between mean 24-h temperature and probability of survival. The default relationship is based on the InSTREAM model values that were developed in lab studies (Railsback et al., 2009).

### Net energy loss

During upstream migration, adult salmon and steelhead are dependent on their initial energy stores for the duration of the migration. The bioenergetics component of the simulation tracked hourly energy use per individual. To evaluate the influence of cold-water refuge use on energy use, we compared differences in mean energy use per population across thermalscapes with and without available CWRs. Energy use can also influence survival. If an individual’s energy density fell below 4 kJ/g, then mortality was imposed as per Crossin et al. (2004).

### Survival uncertainty analysis

Uncertainty in model outcomes can originate from imprecision in parameter estimates, stochasticity within the modeled system, model structure, or uncertainty in future scenarios (Cartwright et al., 2016); our model contained all these sources of uncertainty. Instead of undertaking a protracted and arduous sensitivity analysis of uncertainties in prediction, parameter, and model structure, which we acknowledge could ultimately show large uncertainty around emergent model outcomes, we undertook a limited uncertainty analysis. Our limited uncertainty analysis was to demonstrate areas where increased information could be used to further refine the model. We largely do not report measures of prediction uncertainty associated with simulated fish condition model outcomes because our objective was not to provide precise predictions but rather to simulate key processes and illustrate the magnitude of relative costs of migration that cannot be easily tested in the field or laboratory. We examined two different facets of the model, acute temperature stress, which directly impacts fish survival, and thermoregulatory behavior, which indirectly affects fish condition. For more details on uncertainty analyses, see Appendix S3.

## RESULTS

### Migratory phenotypes

**FIGURE 4:**
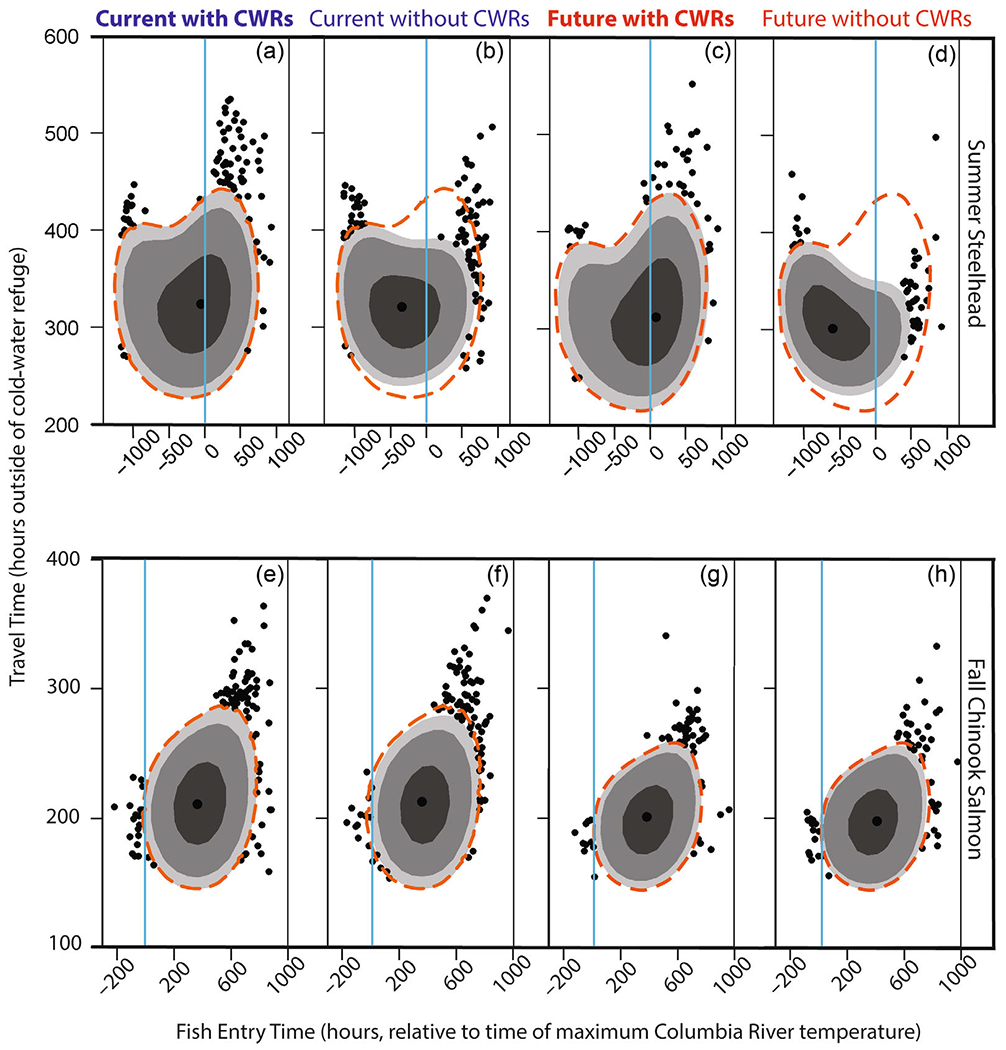
Modeled phenotypic space for adult summer steelhead (a–d) and fall Chinook Salmon (f–h). The shaded regions represent probability of coverage (50% dark gray, 90% medium gray, or 95% light gray) for the energy-conserving (defined here as losing <25% percentile of energy) individuals migrating for that particular combination of migratory phenotypes. Black points represent simulated individuals at value combinations outside of the 95% probability of coverage, excluding the center black point which represents the mode value. Entry timing is presented as relative to model hour maximum Columbia River temperature by subtracting individual entry hour from the model hour of maximum Columbia River temperature. Model hour maximum Columbia River temperature is illustrated by the blue lines. The orange dashed line signifies the 95% probability of coverage from the current scenario with cold-water refuges (CWRs).

**FIGURE 5:**
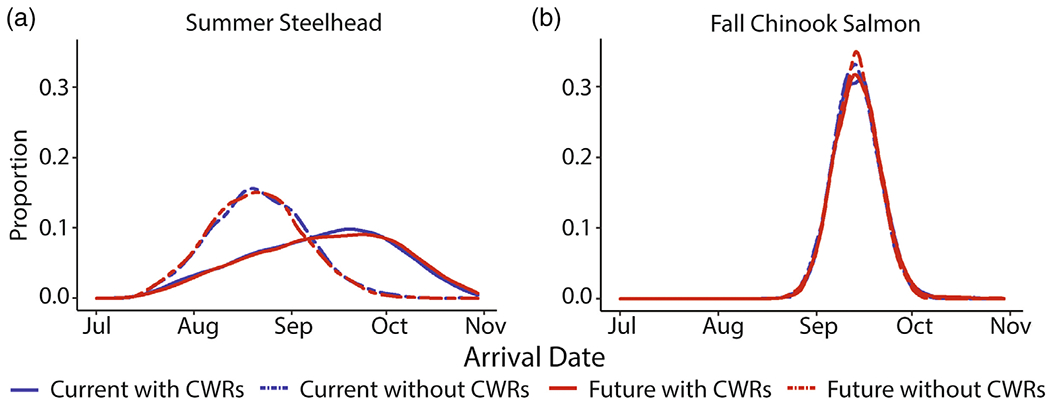
Smoothed histograms of modeled arrival timing at the Columbia River–Snake River confluence for (a) Grande Ronde River Summer Steelhead and (b) Snake River fall Chinook Salmon populations across four thermalscapes: Columbia River current temperatures with access to cold-water refuges (CWRs), Columbia River current temperatures without access to CWRs and Columbia River predicted future (year 2040) temperatures with and without access to CWRs.

Cold-water refuge availability increased the diversity of energy-conserving migratory phenotypes (Figure 4) and the range of arrival timing at the migration corridor terminus (Figure 5). We illustrate the constriction and expansion of the portion of phenotypic space corresponding to energy-conserving migration outcomes by depicting them as a function of a subset of the phenotypic variables defined by travel time outside of CWRs and entry time in relation to maximum Columbia River temperature (Figure 4). At the individual scale, CWRs created opportunities for a greater diversity of migratory phenotypes to exhibit successful energy conservation during migration. Energy-conserving phenotypic space, as illustrated by shaded region in Figure 4, contracted by 23% (steelhead) and 7% (fall Chinook Salmon) when CWRs were unavailable under the current thermal regime. Without CWRs, the majority of energy-conserving steelhead individuals shift to an earlier migration start date in relation to the time of maximum Columbia River water temperatures. Fall Chinook Salmon energy-conserving phenotypic space decreases by 25% based on warmer mainstem temperatures alone. By contrast, warmer mainstem temperatures alone did not change the energy-conserving phenotypic area for steelhead. For steelhead, arrival timing at the migration corridor terminus under current and hypothesized future conditions without available CWRs was constricted, with the majority of individuals arriving in August (Figure 5). This contrasts with thermalscapes that included CWRs, for which the majority of individuals arrived between August and mid-October (Figure 5a).

### Acute and accumulated exposure

**FIGURE 6:**
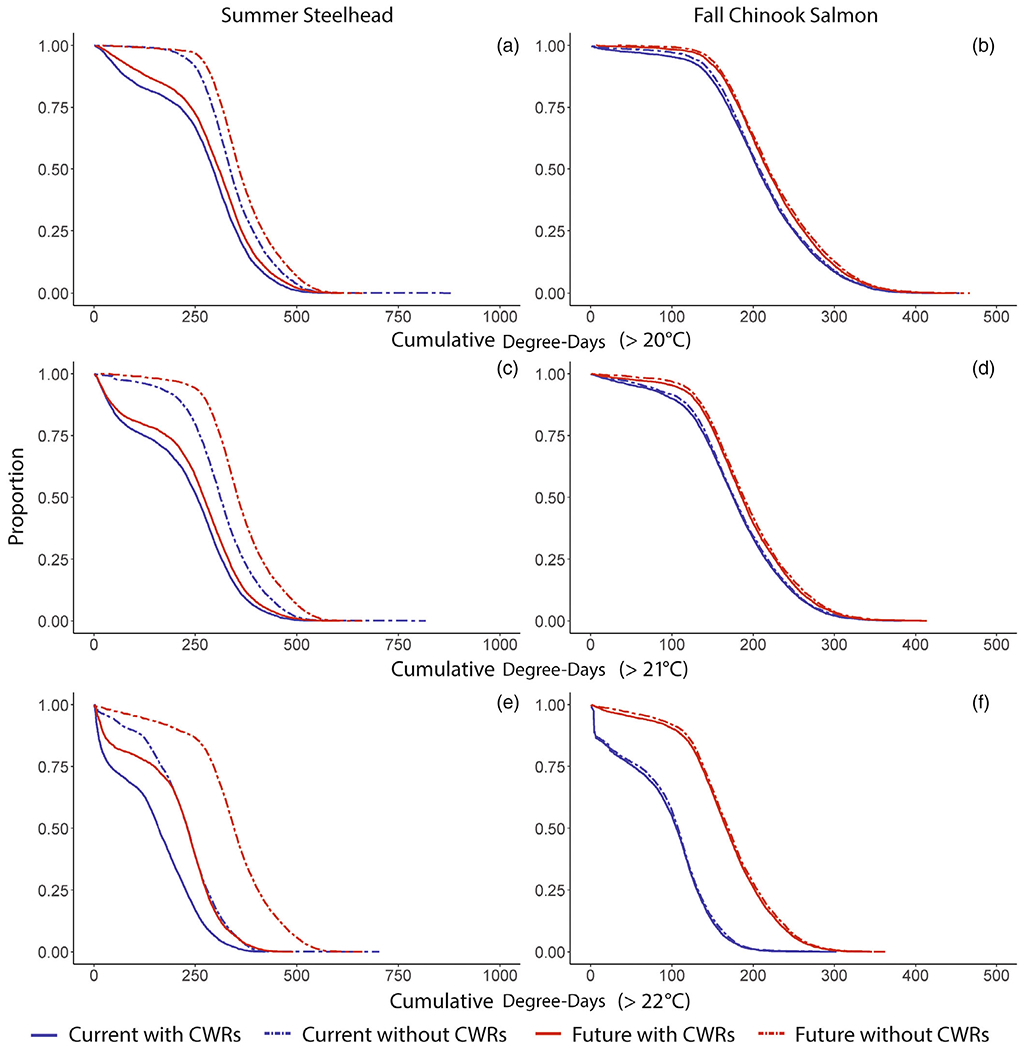
For each modeled scenario, a reverse cumulative distribution function (1-CDF) showing the proportion of individuals within simulated fish populations that have cumulative degree-day exposures exceeding at 20, 21, and 22°C thresholds. (a), (c), and (e) are derived from modeled temperature exposure time series for Grande Ronde River summer steelhead. (b), (d), and (f) are from Snake River fall Chinook Salmon. Cumulative degree-days are calculated as the sum of the hourly temperatures from a modeled individual’s temperature exposure time series, when temperature is above the thresholds (e.g., 20°C). Hourly sums are converted to days by dividing by 24. CWRs, cold-water refuges.

At the population scale, modeled cold-water refuge use frequently decreased accumulated sublethal thermal exposure resulting from warm mainstem temperatures (Figure 6). As the threshold temperature increased, cold-water refuge use became more effective at reducing the proportion of the population with higher accumulated degree-days. For steelhead, behavioral thermoregulation was an effective strategy for decreasing accumulated thermal exposure in both present and future Columbia River conditions. By contrast, fall Chinook Salmon cumulative thermal exposure was influenced largely by Columbia River temperatures and less so by cold-water refuge use. For steelhead, accumulated exposure, observed under current and future Columbia River thermalscapes, and measured at 20, 21, and 22 degree-day thresholds, was markedly lower when CWRs were available (Figure 6, difference between solid and dotted lines). For fall Chinook Salmon, availability of CWRs also decreased chronic exposure at 20, 21, and 22 degree-day thresholds, but by much less than for summer steelhead.

Under current and future thermalscapes, cold-water refuge use has little influence on either Chinook Salmon or steelhead mortality from acute temperature stress. Mortality from acute temperature stress ranged from 0.2% to 0.5% and 1.1% to 1.9% for current and future thermalscapes, respectively.

### Net energy loss

Although we found cold-water refuge use decreased acute thermal exposures, it did not mitigate energy consumption under the current warm mainstem Columbia River thermalscape (Table 2). Our simulations indicated little difference between the mean percent energy used per population under current Columbia River temperatures (Chinook Salmon 19.6% and steelhead 28.8%) compared with simulations with no available CWRs (19.6% and 28.1%, respectively). These model outcomes persisted under hypothesized future warmer thermal regimes for Chinook Salmon (21.6% with CWRs and 21.7% without CWRs) and steelhead (31.2% with CWRs and 31.3% without CWRs). However, future warmer thermalscapes demonstrated an increase in energy used by 2.4%–3.1% for steelhead and 2%–2.1% for Chinook Salmon when compared with the current thermalscape. Additionally, little difference was indicated in variability around the means or by the 25th and 75th percentile distributions (Table 2).

## DISCUSSION

This study provides insights into the importance of CWRs for migrating salmon and steelhead employing behavioral thermoregulation to cope with climate and landscape change. Fitness outcomes emerging from our simulations provided evidence that CWRs can be beneficial to upstream-migrating salmon and steelhead by increasing the diversity of energy-conserving migratory phenotypes, and by reducing the proportion of the population exposed to higher degree-day accumulations. Although we expected behavioral thermoregulation to decrease energy use and increase survival, our model results provide little evidence supporting these outcomes.

Other researchers have noted the importance of portfolio effects, and specifically that increasing the diversity of phenotypic expression decreases variance in adult salmon return abundance across broad spatial extents (Carlson & Satterthwaite, 2011; Greene et al., 2010; Schindler et al., 2010). Collectively, this work suggests that local phenotypic diversity contributes to regional stability in the annual returns of migratory salmon. Maintaining diverse life history portfolios may be essential for populations to be resilient in landscapes altered by climate change (Moore et al., 2014). If results from other studies of portfolio effects in salmon apply to our study populations, the expanded diversity of energy-conserving migratory phenotypes associated with CWRs could be vital to future salmon and steelhead migrations.

Conflicting selection pressures across different life stages are an important component of ecological portfolios (Schluter et al., 1991). In the system studied here, the consequences of early migration could affect other aspects of the life cycle. For example, our simulations suggest that extensive cold-water refuge use by summer steelhead, which are spring spawners with a protracted migration, delays their arrival at the Snake River confluence (Figure 5). Such delays could have consequences for mate selection (McMillan et al., 2007), competition for nest sites (Orcutt et al., 1968), and may affect spawn timing and the emergence of juveniles (Campbell et al., 2019; Chandler & Bjornn, 1988). More immediate consequences of extended cold-water refuge use by summer steelhead could include exposure to angling impacts or natural predators, crowding and disease risk, declines in gamete viability, and other stressors not accounted for in our model (Connor et al., 2019; Keefer et al., 2009; Naughton et al., 2011).

Thermal refuges offer ectothermic species opportunities to modify energy expenditures and to minimize exposures to potentially lethal temperatures. Exposure of Pacific salmonids to acutely high temperatures (>20°C) can lead to lethal physiological stress, increase individual susceptibility to pathogens (Crossin et al., 2008; Fenkes et al., 2016; Mathes et al., 2010), and accelerate embryo loss (Connor et al., 2019; Mann, 2007). Access to thermal refuges reduced acute thermal exposure events for summer steelhead, which begin migration prior to and during the onset of summer maximum temperatures. Later migrating fall Chinook Salmon were less sensitive to availability of thermal refuges. For both species, acute mortality from very high thermal exposures was predicted to be low (<1%) because mainstem temperatures did not approach upper incipient lethal levels (Richter & Kolmes, 2005) in our present-day or future warming scenarios.

Our study focused on upstream-migrating adult Pacific salmon on route to their spawning grounds in the headwaters of the Columbia River basin. Other investigators have characterized these migrants’ use of CWRs as an adaptive response to warming mainstem river temperatures (Berman & Quinn, 1991; High et al., 2006; Keefer et al., 2009, 2019). Our simulation results expand upon these conclusions by suggesting that cold-water refuge use may not provide unconditional benefits. Our individual-based modeling results suggest that cold-water refuge use was not able to mitigate overall increases in energy consumption resulting from warmer mainstem Columbia River temperatures. As hypothesized, migration phenology precluded some individuals from using CWRs as an effective means for mitigating increased energy usage at warmer temperatures. Individuals that began upstream migration prior to the onset of summer maximum temperatures, and who used thermal refuges, spent more time within the migratory corridor and incurred greater energy losses (Figure 1b). CWRs provided the most benefit to fish that emerged from them during the downward-trending portion of the seasonal thermograph (Figure 1d). Cold-water refuge use increased the duration of time spent in the migration corridor, and therefore could increase cumulative energy use even though the rate of energy loss decreased at lower temperatures. This finding adds important nuances to other studies that have examined energy loss in relation to migration duration and temperature (Keefer et al., 2018; Mann et al., 2009; Mesa & Magie, 2006; Siegel et al., 2021). Upriver spawning populations experience strong selective pressure, and many other traits are likely linked to spawning success as well. Other studies have illustrated a variety of ways that long-distance migrants optimize energy use, including strong philopatry, which enables physiological adaptation to local thermal and hydrologic regimes (Farrell et al., 2008; Keefer & Caudill, 2014), optimized migration timing (Crossin et al., 2004; Crozier et al., 2011; Plumb, 2018; Quinn & Adams, 1996) and efficient swimming (e.g., use of hydraulic features) and swim speeds (e.g., metabolically optimum) (Hinch & Rand, 2000; Weihs, 1974). While our study does not provide sufficient basis for making generalizations about behavioral thermoregulation, our results do suggest that other elements of behavior (e.g., migration timing vs. migration duration) may be as important as refuge use in establishing the capacity of organisms to respond to climate change.

### Uncertainties and future recommendations

The simulation model contains uncertainties in model parameters and model structure in addition to stochasticity within the system (e.g., interannual variability in temperature, discharge, dam operations, etc.). The significance of these uncertainties was partially mitigated by our focus on the relative fish condition outcomes associated with general categories of thermalscape changes. It is worth mentioning some key uncertainties for potential research avenues and future applications. Uncertainties in the bioenergetics model include an averaged gonad energy allocation, size- and temperature-related activity coefficients parameterized for other populations, activity costs not accounting for water velocity, ignored metabolic power demands, and simplified energetic accounting for high intensity swimming bouts involved in dam passage. Furthermore, the bioenergetics sub-model did not account for differences in habitat quality (e.g., dissolved oxygen levels or biological interactions) beyond thermal heterogeneity, nor did it include the dynamic conditions of the habitat corridor (e.g., river discharge or velocity). For example, future riverscapes were simulated using a straightforward increase in overall temperatures, and did not incorporate changes in precipitation, snow melt timing, or river discharge in any capacity. Additionally, we based the model thermalscapes on temperature observations taken during the year 2017, which had a relatively high number of days with acutely stressful temperatures for salmon and steelhead in order to maximize the degree to which behavioral thermoregulation is currently occurring and its coincident advantages and disadvantages. However, as the thermograph diverges from typical years and extreme temperature and drought conditions may become more common (e.g., early warming in 2015), the system could potentially reach a tipping point that could change behaviors, model assumptions, model parameters, and interpretation of results (Steel et al., 2019; Williams et al., 2022).

Our uncertainty analysis suggests that, as river temperatures continue to warm, survival will become increasingly sensitive to the shape and effect size of the relationship between temperature and mortality (Appendix S3). CWRs may reduce mortality rates as temperature increases, as seen in the relationship between temperature and mortality, and our uncertainty analysis suggests refuges could be important in decreasing mortality from acute temperature stress. Confidence in our model results could be improved with an enhanced understanding of how fish navigate to CWRs, how they determine when to use and when to exit a refuge, and what drives individual movement behavior during refuge use. To this end, we included some experimental modifications of behavioral thermoregulation in our uncertainty analysis. When we modified individual propensity for behavioral thermoregulation, making it more closely tied to the mainstem Columbia seasonal temperature regime, energy loss decreased with cold-water refuge use. However, this outcome did not emerge from the behaviors we believe are most important. Rather, these hypothetical behavioral phenotypes exhibited lower total mean energy loss, suggesting that the model design did not preclude a more significant beneficial role for CWRs. Uncertainty in parameter estimates and model structure do not substantially inhibit our ability to predict that CWRs are beneficial for upstream-migrating salmon and trout in two key aspects: they increase the diversity of energy-conserving phenotypes and they decrease accumulated thermal exposure for upstream-migrating salmon and steelhead. A useful next step would be to model the effects of cold-water refuge use in the lower Snake River or upper Columbia River portions of the adult upstream migration. Our modeling approach is most suitable for illustrating how, under both current and future temperature regimes, behavioral thermoregulation could influence the migration success of populations of salmon and steelhead trout in highly modified river systems.

## CONCLUSIONS

The general importance of life history and landscape diversity has been long appreciated in ecology and evolution (den Boer, 1968; Southwood, 1988; Wright, 1931). More recently, these ideas have coalesced into the notion of ecological and evolutionary portfolios (Schindler et al., 2015). Development of the portfolio concept was fueled by observations of species such as Pacific salmon that exhibit complex life histories and occupy diverse and dynamic landscapes (Schindler et al., 2010). Similar insights have influenced the evolution of thinking about refuges within the context of climate refugia (Morelli et al., 2016; Reside et al., 2019). Some have argued that refuges generally are an underappreciated factor driving a wide array of ecological and evolutionary processes (Berryman et al., 2006). While these concepts are highly germane to theory in ecology and conservation, empirical datasets with spatial and temporal domains sufficient to test them are rarely available. We therefore made use of a spatially explicit, individual-based, mechanistic–probabilistic model to develop simulated datasets that were adequate for examining the efficacy of refuges and the emergence of portfolio effects. By evaluating a sequence of energetic, survival, and phenology consequences of the use of thermal refuges, we were able to identify mechanisms linking these condition metrics and illustrate how such feedback might generate conflicting selection pressures affecting multiple life cycle elements (Schluter et al., 1991). We observed that the ability of a landscape feature to function as a refuge depends on the context in which it is used and how it contributes to the whole population. Migration involves sequences of events occurring at different times and places, and can thus be challenging to understand (Dingle, 2014; Nathan et al., 2008). Each event along a migratory pathway can impose unique selective consequences on individuals moving between locations. Migration typically involves large energy expenditures and exposes individuals to external physical and biological stressors. Because our mechanistic spatially explicit IBM was parameterized and validated using empirical observations, we were able to get closer to accounting for the biological, ecological, and anthropogenic processes that influenced population outcomes. Such outcomes would be difficult to address with other single approaches such as manipulative experiments. As such, our study generates novel insights into the complex benefits and costs of thermal refuge use and illustrates their variable importance for migratory species in a rapidly changing world.

## Supplementary Material

Supplement1

Supplement2

Supplement3

## Figures and Tables

**TABLE 1:** Estimated volumes and mean and range of water temperatures for the nine identified cold-water refuges simulated in the model.

Cold-water refuge	Total volume (m^3^)	Temperature (°C)	
		Mean	Range
Eagle Creek	2988	13.9	7.8–20.3
Rock Creek	1708	15.4	7.8–20.9
Herman Creek	169,698	10.6	6.3–13.4
Wind River	105,220	13.2	7.3–17.1
Little White Salmon River	1,101,126	8	6.3–10.1
White Salmon River	153,529	11.6	6.9–14.3
Klickitat River	222,029	13.8	6.7–18.5
Deschutes River	880,124	16.8	11.1–21.5
Umatilla River	46,299	18.4	9.5–24.1

**TABLE 2:** Modeled outcomes for mean percent energy used, SD of the mean (in parentheses), 10th and 90th percentiles by population, and modeled thermalscape with and without cold-water refuges (CWRs).

Thermalscape	Population	Percent energy used	25th percentile	75th percentile
Current, CWRs available	Summer steelhead	28.8 (±5.5)	24.9	32.4
Current, CWRs unavailable	Summer steelhead	28.1 (±5.4)	24.2	31.6
Future, CWRs available	Summer steelhead	31.2 (±5.9)	27.0	35.0
Future, CWRs unavailable	Summer steelhead	31.3 (±6.0)	26.9	35.1
Current, CWRs available	Fall Chinook Salmon	19.6 (±4.4)	16.3	22.5
Current, CWRs unavailable	Fall Chinook Salmon	19.6 (±4.4)	16.4	22.4
Future, CWRs available	Fall Chinook Salmon	21.6 (±4.8)	17.9	24.8
Future, CWRs unavailable	Fall Chinook Salmon	21.7 (±4.9)	18.0	25.0

## Data Availability

R code used to summarize model results (Snyder, 2022) is available from Zenodo: https://zenodo.org/badge/latestdoi/518287795. The parameterized model and outcomes used to generate results for each experiment (Ebersole, 2019) are available from the US EPA’s Environmental Dataset Gateway: https://doi.org/10.23719/1503532.

## References

[R1] Beever EA, Embere Hall L, Varner J, Loosen AE, Dunham JB, Gahl MK, Smith FA, and Lawler JJ. 2017. “Behavioral Flexibility as a Mechanism for Coping with Climate Change.” Frontiers in Ecology and the Environment 15(6): 299–308.

[R2] Berman CH, and Quinn TP. 1991. “Behavioural Thermoregulation and Homing by Spring Chinook Salmon, *Oncorhynchus tshawytscha* (Walbaum), in the Yakima River.” Journal of Fish Biology 39(3): 301–12.

[R3] Berryman AA, Hawkins BA, and Hawkins BA. 2006. “The Refuge as an Integrating Concept in Ecology and Evolution.” Oikos 115(1): 192–6.

[R4] Bottom DL, Jones KK, Simenstad CA, and Smith CL. 2009. “Reconnecting Social and Ecological Resilience in Salmon Ecosystems.” Ecology and Society 14(1): 5.

[R5] Bowerman TE, Pinson-Dumm A, Peery CA, and Caudill CC. 2017. “Reproductive Energy Expenditure and Changes in Body Morphology for a Population of Chinook Salmon Oncorhynchus tshawytscha with a Long Distance Migration.” Journal of Fish Biology 90(5): 1960–79. 28211057 10.1111/jfb.13274

[R6] Brown RS, Geist DR, and Mesa MG. 2006. “Use of Electromyogram Telemetry to Assess Swimming Activity of Adult Spring Chinook Salmon Migrating Past a Columbia River Dam.” Transactions of the American Fisheries Society 135(2): 281–7.

[R7] Campbell EY, Dunham JB, Reeves GH, and Wondzell SM. 2019. “Phenology of Hatching, Emergence, and End-of-Season Body Size in Young-of-Year Coho Salmon in Thermally Contrasting Streams Draining the Copper River Delta, Alaska.” Canadian Journal of Fisheries and Aquatic Sciences 76(2): 185–91.

[R8] Carlson SM, and Satterthwaite WH. 2011. “Weakened Portfolio Effect in a Collapsed Salmon Population Complex.” Canadian Journal of Fisheries and Aquatic Sciences 68(9): 1579–89.

[R9] Cartwright SJ, Bowgen KM, Collop C, Hyder K, Nabe-Nielsen J, Stafford R, Stillman RA, Thorpe RB, and Sibly RM. 2016. “Communicating Complex Ecological Models to Non-scientist End Users.” Ecological Modelling 338: 51–9.

[R10] Chandler GL, and Bjornn TC. 1988. “Abundance, Growth, and Interactions of Juvenile Steelhead Relative to Time of Emergence.” Transactions of the American Fisheries Society 117(5): 432–43.

[R11] Connor WP, Tiffan KF, Chandler JA, Rondorf DW, Arnsberg BD, and Anderson KC. 2019. “Upstream Migration and Spawning Success of Chinook Salmon in a Highly Developed, Seasonally Warm River System.” Reviews in Fisheries Science & Aquaculture 27(1): 1–50.

[R12] Conrad RH, and Gutmann JL. 1996. “Conversion Equations between Fork Length and Total Length for Chinook Salmon (*Oncorhynchus tshawytscha*).” Northwest Indian Fisheries Commission. Project Report Series No. 5, 40 pp.

[R13] Crossin GT, Hinch SG, Cooke SJ, Welch DW, Patterson DA, Jones SRM, Lotto AG, 2008. “Exposure to High Temperature Influences the Behaviour, Physiology, and Survival of Sockeye Salmon during Spawning Migration.” Canadian Journal of Zoology 86(2): 127–40.

[R14] Crossin GT, Hinch SG, Farrell AP, Higgs DA, Lotto AG, Oakes JD, and Healey MC. 2004. “Energetics and Morphology of Sockeye Salmon: Effects of Upriver Migratory Distance and Elevation.” Journal of Fish Biology 65(3): 788–810.

[R15] Crozier LG, Bowerman TE, Burke BJ, Keefer ML, and Caudill CC. 2017. “High-Stakes Steeplechase: A Behavior-Based Model to Predict Individual Travel Times through Diverse Migration Segments.” Ecosphere 8(10): e01965.

[R16] Crozier LG, McClure MM, Beechie T, Bograd SJ, Boughton DA, Carr M, Cooney TD, 2019. “Climate Vulnerability Assessment for Pacific Salmon and Steelhead in the California Current Large Marine Ecosystem.” PloS One 14(7): e0217711. 31339895 10.1371/journal.pone.0217711 PMC6655584

[R17] Crozier LG, Scheuerell MD, and Zabel RW. 2011. “Using Time Series Analysis to Characterize Evolutionary and Plastic Responses to Environmental Change: A Case Study of a Shift toward Earlier Migration Date in Sockeye Salmon.” The American Naturalist 178(6): 755–73. 10.1086/662669 22089870

[R18] Davis J, Pavlova A, Thompson R, and Sunnucks P. 2013. “Evolutionary Refugia and Ecological Refuges: Key Concepts for Conserving Australian Arid Zone Freshwater Biodiversity under Climate Change.” Global Change Biology 19(7): 1970–84. 23526791 10.1111/gcb.12203 PMC3746109

[R19] den Boer PJ 1968. “Spreading of Risk and Stabilization of Animal Numbers.” Acta Biotheoretica 18(1): 165–94. 4984481 10.1007/BF01556726

[R20] Deslauriers D, Chipps SR, Breck JE, Rice JA, and Madenjian CP. 2017. “Fish Bioenergetics 4.0: An R-Based Modeling Application.” Fisheries 42(11): 586–96.

[R21] Dingle H 2014. Migration: The Biology of Life on the Move 340. USA: Oxford University Press.

[R22] Ebersole JL 2019. “Migration Corridor Simulation.” US Environmental Protection Agency. 10.23719/1503532.

[R23] Ebersole JL, Quiñones RM, Clements S, and Letcher BH. 2020. “Managing Climate Refugia for Freshwater Fishes under an Expanding Human Footprint.” Frontiers in Ecology and the Environment 18(5): 271–80. 32944010 10.1002/fee.2206 PMC7490791

[R24] Farrell AP, Hinch SG, Cooke SJ, Patterson DA, Crossin GT, Lapointe M, and Mathes MT. 2008. “Pacific Salmon in Hot Water: Applying Aerobic Scope Models and Biotelemetry to Predict the Success of Spawning Migrations.” Physiological and Biochemical Zoology 81(6): 697–708. 18922081 10.1086/592057

[R25] Fausch KD, Torgersen CE, Baxter CV, and Li HW. 2002. “Landscapes to Riverscapes: Bridging the Gap between Research and Conservation of Stream Fishes.” BioScience 52(6): 483.

[R26] Fenkes M, Shiels HA, Fitzpatrick JL, and Nudds RL. 2016. “The Potential Impacts of Migratory Difficulty, Including Warmer Waters and Altered Flow Conditions, on the Reproductive Success of Salmonid Fishes.” Comparative Biochemistry and Physiology. Part A, Molecular & Integrative Physiology 193: 11–21. 10.1016/j.cbpa.2015.11.012 PMC4751979 26603555

[R27] Fey SB, Vasseur DA, Alujević K, Kroeker KJ, Logan ML, O’Connor MI, Rudolf VHW, 2019. “Opportunities for Behavioral Rescue under Rapid Environmental Change.” Global Change Biology 25(9): 3110–20. 31148329 10.1111/gcb.14712

[R28] Frechette DM, Dugdale SJ, Dodson JJ, and Bergeron NE. 2018. “Understanding Summertime Thermal Refuge Use by Adult Atlantic Salmon Using Remote Sensing, River Temperature Monitoring, and Acoustic Telemetry.” Canadian Journal of Fisheries and Aquatic Sciences 75(11): 1999–2010.

[R29] Goniea TM, Keefer ML, Bjornn TC, Peery CA, Bennett DH, and Stuehrenberg LC. 2006. “Behavioral Thermoregulation and Slowed Migration by Adult Fall Chinook Salmon in Response to High Columbia River Water Temperatures.” Transactions of the American Fisheries Society 135(2): 408–19.

[R30] Greene CM, Hall JE, Guilbault KR, and Quinn TP. 2010. “Improved Viability of Populations with Diverse Life-History Portfolios.” Biology Letters 6(3): 382–6. 20007162 10.1098/rsbl.2009.0780 PMC2880035

[R31] Groves PA, and Chandler JA. 1999. “Spawning Habitat Used by Fall Chinook Salmon in the Snake River.” North American Journal of Fisheries Management 19(4): 912–22.

[R32] High B, Peery CA, and Bennett DH. 2006. “Temporary Staging of Columbia River Summer Steelhead in Coolwater Areas and its Effect on Migration Rates.” Transactions of the American Fisheries Society 135(2): 519–28.

[R33] Hinch SG, and Rand PS. 2000. “Optimal Swimming Speeds and Forward-Assisted Propulsion: Energy-Conserving Behaviours of Upriver-Migrating Adult Salmon.” Canadian Journal of Fisheries and Aquatic Sciences 57(12): 2470–8.

[R34] IPCC. 2019. “Climate Change and Land: An IPCC Special Report on Climate Change, Desertification, Land Degradation, Sustainable Land Management, Food Security, and Greenhouse Gas Fluxes in Terrestrial Ecosystems.” Edited by Shukla PR, Skea J, Calvo Buendia E, Masson-Delmotte V, Pörtner HO, Roberts DC, Zhai P, Slade R, Connors S, van Diemen R, and Ferrat M, 874.

[R35] Isaak DJ, Wenger SJ, Peterson EE, Ver Hoef JM, Nagel DE, Luce CH, Hostetler SW, 2017. “The NorWeST Summer Stream Temperature Model and Scenarios for the Western US: A Crowd-Sourced Database and New Geospatial Tools Foster a User Community and Predict Broad Climate Warming of Rivers and Streams.” Water Resources Research 53(11): 9181–205.

[R36] Isaak DJ, Wollrab S, Horan D, and Chandler G. 2012. “Climate Change Effects on Stream and River Temperatures across the Northwest US from 1980–2009 and Implications for Salmonid Fishes.” Climatic Change 113(2): 499–524.

[R37] Isaak DJ, Young MK, Luce CH, Hostetler SW, Wenger SJ, Peterson EE, Ver Hoef JM, Groce MC, Horan DL, and Nagel DE. 2016. “Slow Climate Velocities of Mountain Streams Portend their Role as Refugia for Cold-Water Biodiversity.” Proceedings of the National Academy of Sciences of the United States of America 113(16): 4374–9. 27044091 10.1073/pnas.1522429113 PMC4843441

[R38] Jager HI 2011. “Quantifying Temperature Effects on Fall Chinook Salmon.” Oak Ridge, TN: US Department of Energy, Oak Ridge National Laboratory, 10, 1047614.

[R39] Jepson MA, Keefer ML, Naughton GP, Peery CA, and Burke BJ. 2010. “Population Composition, Migration Timing, and Harvest of Columbia River Chinook Salmon in Late Summer and Fall.” North American Journal of Fisheries Management 30(1): 72–88.

[R40] Kearney M, and Porter W. 2009. “Mechanistic Niche Modelling: Combining Physiological and Spatial Data to Predict species’ Ranges.” Ecology Letters 12(4): 334–50. 19292794 10.1111/j.1461-0248.2008.01277.x

[R41] Kearney MR, Wintle BA, and Porter WP. 2010. “Correlative and Mechanistic Models of Species Distribution Provide Congruent Forecasts under Climate Change.” Conservation Letters 3(3): 203–13.

[R42] Keefer ML, and Caudill CC. 2014. “Homing and Straying by Anadromous Salmonids: A Review of Mechanisms and Rates.” Reviews in Fish Biology and Fisheries 24(1): 333–68.

[R43] Keefer ML, Clabough TS, Jepson MA, Bowerman T, and Caudill CC. 2019. “Temperature and Depth Profiles of Chinook Salmon and the Energetic Costs of their Long-Distance Homing Migrations.” Journal of Thermal Biology 79: 155–65. 30612677 10.1016/j.jtherbio.2018.12.011

[R44] Keefer ML, Clabough TS, Jepson MA, Johnson EL, Peery CA, and Caudill CC. 2018. “Thermal Exposure of Adult Chinook Salmon and Steelhead: Diverse Behavioral Strategies in a Large and Warming River System.” PLoS One 13(9): e0204274. 30240404 10.1371/journal.pone.0204274 PMC6150539

[R45] Keefer ML, Jepson MA, Clabough TS, and Caudill CC. 2021. “Technical Fishway Passage Structures Provide High Passage Efficiency and Effective Passage for Adult Pacific Salmonids at Eight Large Dams.” PloS One 16(9): e0256805. 34473741 10.1371/journal.pone.0256805 PMC8412358

[R46] Keefer ML, Jepson MA, Clabough TS, Caudill CC, Bjornn TC, Burke BJ, Matter AL, Moser ML, Perry CA, and Stuehrenberg LC. 2017. “Migration of Adult Salmonids in the Federal Columbia River Hydrosystem: A Summary of Radiotelemetry Studies, 1996–2014 (No. 2017-2).” US ACOE Portland District.

[R47] Keefer ML, Peery CA, Bjornn TC, Jepson MA, and Stuehrenberg LC. 2004. “Hydrosystem, Dam, and Reservoir Passage Rates of Adult Chinook Salmon and Steelhead in the Columbia and Snake Rivers.” Transactions of the American Fisheries Society 133(6): 1413–39.

[R48] Keefer ML, Peery CA, and High B. 2009. “Behavioral Thermoregulation and Associated Mortality Trade-Offs in Migrating Adult Steelhead (*Oncorhynchus mykiss*): Variability among Sympatric Populations.” Canadian Journal of Fisheries and Aquatic Sciences 66(10): 1734–47.

[R49] Lindström Å, Alerstam T, Bahlenberg P, Ekblom R, Fox JW, Råghall J, and Klaassen RH. 2016. “The Migration of the Great Snipe *Gallinago media*: Intriguing Variations on a Grand Theme.” Journal of Avian Biology 47(3): 321–34.

[R50] Mahmoudi R, Soltani M, Matinfar A, Rezvani Gilkolai S, and Abolghasem K. 2014. “Morphometric Relationship between Length-Weight, Length-Length, and Condition Factor in Farmed Rainbow Trout (*Oncorhynchus mykiss*).” Bulletin of Environment, Pharmacology and Life Sciences 3(4): 215–20.

[R51] Mann RD 2007. “The Effects of High Temperature Exposures on Migration Success and Embryo Quality of Snake River Adult Chinook Salmon and Steelhead (Master’s).” University of Idaho.

[R52] Mann RD, Peery CA, Pinson AM, and Anderson CR. 2009. “Energy Use, Migration Times, and Spawning Success of Adult Spring–Summer Chinook Salmon Returning to Spawning Areas in the South Fork Salmon River in Central Idaho: 2002–2007 (No. 2009-4).” Idaho Cooperative Fish and Wildlife Research Unit, University of Idaho.

[R53] Mathes MT, Todd Mathes M, Hinch SG, Cooke SJ, Crossin GT, Patterson DA, Lotto AG, and Farrell AP. 2010. “Effect of Water Temperature, Timing, Physiological Condition, and Lake Thermal Refugia on Migrating Adult Weaver Creek Sockeye Salmon (*Oncorhynchus nerka*).” Canadian Journal of Fisheries and Aquatic Sciences 67(1): 70–84.

[R54] McCullough DA, Bartholow JM, Jager HI, Beschta RL, Cheslak EF, Deas ML, Ebersole JL, 2009. “Research in Thermal Biology: Burning Questions for Coldwater Stream Fishes.” Reviews in Fisheries Science 17(1): 90–115.

[R55] McMillan JR, Katz SL, and Pess GR. 2007. “Observational Evidence of Spatial and Temporal Structure in a Sympatric Anadromous (Winter Steelhead) and Resident Rainbow Trout Mating System on the Olympic Peninsula, Washington.” Transactions of the American Fisheries Society 136(3): 736–48.

[R56] Mesa MG, and Magie CD. 2006. “Evaluation of Energy Expenditure in Adult Spring Chinook Salmon Migrating Upstream in the Columbia River Basin: An Assessment Based on Sequential Proximate Analysis.” River Research and Applications 22(10): 1085–95.

[R57] Moore JW, Yeakel JD, Peard D, Lough J, and Beere M. 2014. “Life-History Diversity and its Importance to Population Stability and Persistence of a Migratory Fish: Steelhead in Two Large North American Watersheds.” Journal of Animal Ecology 83(5): 1035–46. 24673479 10.1111/1365-2656.12212

[R58] Morelli TL, Daly C, Dobrowski SZ, Dulen DM, Ebersole JL, Jackson ST, Lundquist JD, 2016. “Managing Climate Change Refugia for Climate Adaptation.” PLoS One 11(8): e0159909. 27509088 10.1371/journal.pone.0159909 PMC4980047

[R59] Nathan R, Getz WM, Revilla E, Holyoak M, Kadmon R, Saltz D, and Smouse PE. 2008. “A Movement Ecology Paradigm for Unifying Organismal Movement Research.” Proceedings of the National Academy of Sciences of the United States of America 105(49): 19052–9. 19060196 10.1073/pnas.0800375105 PMC2614714

[R60] National Marine Fisheries Service (NMFS). 2016. “Status of ESA Listings and Critical Habitat Designations for West Coast Salmon and Steelhead, 2016.” National Marine Fisheries Service. Northwest Region. https://www.fisheries.noaa.gov/resource/document/status-esa-listings-and-critical-habitat-designations-west-coast-salmon-and.

[R61] Naughton GP, Keefer ML, Clabough TS, Jepson MA, Lee SR, Peery CA, and Caudill CC. 2011. “Influence of Pinniped-Caused Injuries on the Survival of Adult Chinook Salmon (*Oncorhynchus tshawytscha*) and Steelhead Trout (*Oncorhynchus mykiss*) in the Columbia River Basin.” Canadian Journal of Fisheries and Aquatic Sciences 68(9): 1615–24.

[R62] Orcutt DR, Pulliam BR, and Arp A. 1968. “Characteristics of Steelhead Trout Redds in Idaho Streams.” Transactions of the American Fisheries Society 97(1): 42–5.

[R63] Payne JT, Wood AW, Hamlet AF, Palmer RN, and Lettenmaier DP. 2004. “Mitigating the Effects of Climate Change on the Water Resources of the Columbia River Basin.” Climatic Change 62(1-3): 233–56.

[R64] Plumb JM 2018. “A Bioenergetics Evaluation of Temperature-Dependent Selection for the Spawning Phenology by Snake River Fall Chinook Salmon.” Ecology and Evolution 8(19): 9633–45. 30386563 10.1002/ece3.4353 PMC6202718

[R65] Quinn TP, and Adams DJ. 1996. “Environmental Changes Affecting the Migratory Timing of American Shad and Sockeye Salmon.” Ecology 77(4): 1151–62.

[R66] Railsback SF, Harvey BC, Jackson SK, and Lamberson RH. 2009. “InSTREAM: The Individual-Based Stream Trout Research and Environmental Assessment Model.” Gen. Tech. Rep. PSW-GTR-218. Albany, CA: US Department of Agriculture, Forest Service, Pacific Southwest Research Station. 254 p, 218.

[R67] Reside AE, Briscoe NJ, Dickman CR, Greenville AC, Hradsky BA, Kark S, Kearney MR, 2019. “Persistence through Tough Times: Fixed and Shifting Refuges in Threatened Species Conservation.” Biodiversity and Conservation 28(6): 1303–30.

[R68] Richter A, and Kolmes SA. 2005. “Maximum Temperature Limits for Chinook, Coho, and Chum Salmon, and Steelhead Trout in the Pacific Northwest.” Reviews in Fisheries Science 13(1): 23–49.

[R69] Rivrud IM, Sivertsen TR, Mysterud A, Åhman B, Støen OG, and Skarin A. 2018. “Reindeer Green-Wave Surfing Constrained by Predators.” Ecosphere 9(5): e02210.

[R70] Robards MD, and Quinn TP. 2002. “The Migratory Timing of Adult Summer-Run Steelhead in the Columbia River over Six Decades of Environmental Change.” Transactions of the American Fisheries Society 131(3): 523–36.

[R71] Sabal MC, Workman ML, Merz JE, and Palkovacs EP. 2021. “Shade Affects Magnitude and Tactics of Juvenile Chinook Salmon Antipredator Behavior in the Migration Corridor.” Oecologia 197(1): 89–100. 34355272 10.1007/s00442-021-05008-4 PMC8445879

[R72] Schindler DE, Armstrong JB, and Reed TE. 2015. “The Portfolio Concept in Ecology and Evolution.” Frontiers in Ecology and the Environment 13(5): 257–63.

[R73] Schindler DE, Hilborn R, Chasco B, Boatright CP, Quinn TP, Rogers LA, and Webster MS. 2010. “Population Diversity and the Portfolio Effect in an Exploited Species.” Nature 465(7298): 609–12. 20520713 10.1038/nature09060

[R74] Schluter D, Price TD, and Rowe L. 1991. “Conflicting Selection Pressures and Life History Trade-Offs.” Proceedings of the Royal Society of London. Series B: Biological Sciences 246(1315): 11–7.

[R75] Schneider JC 2000. “Manual of Fisheries Survey Methods II: With Periodic Updates.” Michigan Department of Natural Resources. Fisheries Special Report 25.

[R76] Schumaker NH, and Brookes A. 2018. “HexSim: A Modeling Environment for Ecology and Conservation.” Landscape Ecology 33: 197–211. 29545713 10.1007/s10980-017-0605-9 PMC5846496

[R77] Sears MW, Angilletta MJ Jr., Schuler MS, Borchert J, Dilliplane KF, Stegman M, Rusch TW, and Mitchell WA. 2016. “Configuration of the Thermal Landscape Determines Thermoregulatory Performance of Ectotherms.” Proceedings of the National Academy of Sciences of the United States of America 113(38): 10,595–600. 27601639 10.1073/pnas.1604824113 PMC5035910

[R78] Seelbach PW 1993. “Population Biology of Steelhead in a Stable-Flow, Low-Gradient Tributary of Lake Michigan.” Transactions of the American Fisheries Society 122(2): 179–98.

[R79] Shamoun-Baranes J, Bouten W, and van Loon EE. 2010. “Integrating Meteorology into Research on Migration.” Integrative and Comparative Biology 50(3): 280–92. 20811515 10.1093/icb/icq011 PMC2931313

[R80] Siegel JE, Crozier LG, Wiesebron LE, and Widener DL. 2021 “Environmentally Triggered Shifts in Steelhead Migration Behavior and Consequences for Survival in the Mid-Columbia River.” PLoS One 16(5): e0250831. 33970924 10.1371/journal.pone.0250831 PMC8109777

[R81] Snyder MN 2022. “Snydermn/CWR_Ecosphere.” R Code (v1.0). Zenodo. 10.5281/zenodo.6910476.

[R82] Snyder MN, Schumaker NH, Ebersole JL, Dunham JB, Comeleo RL, Keefer ML, Leinenbach P, 2019. “Individual Based Modeling of Fish Migration in a 2-D River System: Model Description and Case Study.” Landscape Ecology 34(4): 737–54. 33424124 10.1007/s10980-019-00804-z PMC7788051

[R83] Southwood TRE 1988. “Tactics, Strategies and Templets.” Oikos 52(1): 3.

[R84] Steel EA, Marsha A, Fullerton AH, Olden JD, Larkin NK, Lee S-Y, and Ferguson A. 2019. “Thermal Landscapes in a Changing Climate: Biological Implications of Water Temperature Patterns in an Extreme Year.” Canadian Journal of Fisheries and Aquatic Sciences 76(10): 1740–56.

[R85] Stewart DJ, and Ibarra M. 1991. “Predation and Production by Salmonine Fishes in Lake Michigan, 1978–88.” Canadian Journal of Fisheries and Aquatic Sciences 48(5): 909–22.

[R86] Stouder DJ, Bisson PA, and Naiman RJ. 1997. Pacific Salmon & their Ecosystems: Status and Future Options. Boston, MA: Springer Science & Business Media.

[R87] Sullivan K, Martin DJ, Cardwell RD, Toll JE, and Duke S. 2000. An Analysis of the Effects of Temperature on Salmonids of the Pacific Northwest with Implications for Selecting Temperature Criteria. Portland, OR: Sustainable Ecosystems Institute.

[R88] Torgersen CE, Price DM, Li HW, and McIntosh BA. 1999. “Multiscale Thermal Refugia and Stream Habitat Associations of Chinook Salmon in Northeastern Oregon.” Ecological Applications 9(1): 301–19.

[R89] University of Washington. 2018. “Fish Passage Data.” Columbia River DART (Data Access Real Time). https://www.cbr.washington.edu/dart.

[R90] US Environmental Protection Agency. 2020. “Columbia River Cold Water Refuges.” Available at: https://www.epa.gov/columbiariver/columbia-river-cold-water-refuges.

[R91] US Geological Survey. 2016. “The StreamStats Program.” http://streamstats.usgs.gov.

[R92] US Geological Survey. 2018. “National Hydrography Dataset (ver. USGS National Hydrography Dataset Best Resolution (NHD) for Hydrologic Unit (HU)).” https://www.usgs.gov/core-science-systems/ngp/national-hydrography/access-national-hydrography-products.

[R93] Ween GB, and Colombi BJ. 2013. “Two Rivers: The Politics of Wild Salmon, Indigenous Rights and Natural Resource Management.” Sustainability 5(2): 478–95.

[R94] Weihs D 1974. “Energetic Advantages of Burst Swimming of Fish.” Journal of Theoretical Biology 48(1): 215–29. 4456041 10.1016/0022-5193(74)90192-1

[R95] Wiens JJ 2016. “Climate-Related Local Extinctions Are Already Widespread among Plant and Animal Species.” PLoS Biology 14(12): e2001104. 27930674 10.1371/journal.pbio.2001104 PMC5147797

[R96] Williams AP, Cook BI, and Smerdon JE. 2022. “Rapid Intensification of the Emerging Southwestern North American Megadrought in 2020–2021.” Nature Climate Change 12: 1–3.

[R97] Wright S 1931. “Evolution in Mendelian Populations.” Genetics 16(2): 97–159. 17246615 10.1093/genetics/16.2.97 PMC1201091

[R98] Yearsley JR 2009. “A Semi-Lagrangian Water Temperature Model for Advection-Dominated River Systems.” Water Resources Research 45(12): 1–19.

