# Supplementary material for: Tough places and safe spaces: Can refuges save salmon from a warming climate?
Source: Ecosphere. Author manuscript; Available in PMC 2023 Nov 9. (PMC9728623; doi:10.1002/ecs2.4265)
Supplement: Supplement1 [file NIHMS1850533-supplement-Supplement1.pdf]

Appendix S3. Uncertainty Analysis

Supplementary Information for Ecosphere: Tough places and safe spaces: Can refuges save salmon from a warming climate?

Snyder, Marcía N.[1*], Schumaker, Nathan H.[1], Dunham, Jason B.[2], Ebersole, Joseph L.[1], Keefer, Matthew L.[3], Halama, Jonathan[1,4], Comeleo, Randy L.[1], Leinenbach, Peter[5], Brookes, Allen[1], Cope, Ben[5], Wu, Jennifer[5], Palmer, John[5]

[1]US Environmental Protection Agency, Pacific Ecological Systems Division, 200 SW 35th St., Corvallis, OR 97333

[2]US Geological Survey, Forest and Rangeland Ecosystem Science Center, 3200 SW Jefferson Way, Corvallis, OR 97331

[3]University of Idaho, Department of Fish and Wildlife Sciences, College of Natural Resources, 975 W. Sixth Street, Moscow, Idaho 83844

[4]Oak Ridge Institute for Science and Education/US Environmental Protection Agency, Pacific Ecological Systems Division, 200 SW 35th St., Corvallis, OR 97333

[5]US Environmental Protection Agency, Region 10, 1200 6th Ave., Suite 155, Seattle, WA 98101

*Corresponding author, ORCID: 0000-0003-2202-2668, email: snydermn@gmail.com, phone: 1-541-754-4423

Appendix S3. Uncertainty Analysis

The uncertainty analysis had two objectives: 1) to highlight how uncertainty around acute lethal stress will impact fish survival and 2) to address uncertainty in the movement behavior model structure. Uncertainty analysis was simulated with Grande Ronde River summer steelhead populations.

Acute temperature stress

Acute temperature is a well-defined stressor that can be readily measured in laboratory experiments (e.g., Brett et al. 1952; Sullivan et al. 2000). In contrast, the physiological and survival consequences of sub-lethal temperature stress can be more difficult to discern because thermal exposure interactions with other stressors can be complex and non-additive (Richter and Kolmes 2005; McCullough et al. 2009; von Biela et al. 2020). However, there was uncertainty in the parameter estimates, specifically in how to translate existing experimental endpoints into species- and population-specific relationships between exposure duration and lethality. Our survival uncertainty analysis examined how the uncertainty in translation of the existing experimental endpoints into species specific relationships of exposure duration and lethality could influence modeled survival outcomes. For the test of uncertainty around acute lethal stress we simulated three different acute temperature stress relationships based on default, exponential, or logistic empirical studies (Jager, 2011; Railsback et al., 2009; Sullivan et al., 2000) (Figure S1). We examined how each relationship impacted overall mortality for the current and future thermalscapes with and without cold-water refuges. Acute temperature stress was measured by the percent of simulated individuals experiencing mortality from acute temperature stress.

Uncertainty analyses of acute temperature stress and survival revealed no significant change in survival for current thermalscapes (Table S1). Under the current thermalscape,

mainstem Columbia River temperatures are not often reaching values where acute temperature stress has a large impact. However, simulation forecasts run with a warmer Columbia River exhibited mortalities ranging from 0-28%, indicating potentially important differences among the parameterizations. The point at which acute temperature stress becomes important depends on the shape of the curve. As mainstem Columbia River temperatures increase, simulations suggest higher mortality from acute temperature stress could occur and cold-water refuges could be increasingly important in avoiding mortality from acute temperature stress. The exponential form would have the greatest effect in the near term and the logistic would have the greatest effect at the inflection point.

Movement behavior

The uncertainty analysis objective is to address uncertainty in the movement behavior model structure. We performed a limited uncertainty analysis to understand the potential for limitations in our knowledge of salmonid movement behavior to impact fish condition outcomes. The uncertainty analysis compared changes in two fish condition outcomes, namely survival and mean energy use which are summarized in table form below for each analysis. Acute temperature stress was measured by the same means as the uncertainty analysis examining survival (percent of simulated individuals experiencing mortality from acute temperature stress). Energy use was evaluated by comparing the median percent energy used during the simulated migration for the total population across scenarios.

The limited uncertainty analysis of fish behavior explored how survival and energy use changed when varying a key parameter, the probability of moving to cold-water refuges. We modified the probability of having moved to cold water refuge based on timing of temperature

trends in the Columbia River. By decreasing the probability of moving to cold-water refuges while mainstem Columbia River is warming, increasing the probability during maximum temperatures, and decreasing the probability when temperatures are decreasing, we created risk averse fish movement behavior. For the analysis of uncertainty, the probability of moving to cold water on any given hour was modified based on the model week using a parabola shaped curve. For each uncertainty analysis, we simulated the current thermalscape (based on year 2017) with cold-water refuges for the Grande Ronde River summer steelhead population. Simulations modified the move to cold water refuge probability based on the seasonal thermograph for three different factors of increasing strength: A, B, and C. The factors increased the hourly probability of moving to cold-water refuge at the hottest temperature during the peak seasonal thermograph by 18-227%. The hourly probability of moving to cold-water refuge at the maximum temperature during the earliest and latest weeks of migration was decreased between 19-100% probability.

Small changes in mortality and energy use were observed in the uncertainty analyses examining fish behavior, for which cold-water refuge use was modified based on the relationship to the mainstem Columbia River seasonal temperatures (Table S2). This analysis demonstrates that when behavioral thermoregulation was modified to be more closely tied to the mainstem Columbia seasonal temperature regime, energy loss decreased with cold-water refuge use. This effect was more pronounced as the effect strength increased from A to C.

Literature Cited

Brett, J. R. (1952). Temperature tolerance in young Pacific Salmon, genus *Oncorhynchus. Journal of the Fisheries Board of Canada, 9*(6),265-323.

Jager, H. I. (2011). Quantifying temperature effects on fall Chinook salmon. *Oak Ridge, TN: US Department of Energy, Oak Ridge National Laboratory*, *10*, 1047614.

McCullough, D. A., Bartholow, J. M., Jager, H. I., Beschta, R. L., Cheslak, E. F., Deas, M. L., ... & Wurtsbaugh, W. A. (2009). Research in thermal biology: burning questions for coldwater stream fishes. *Reviews in Fisheries Science*, *17*(1), 90-115.

Railsback, S. F., Harvey, B. C., Jackson, S. K., & Lamberson, R. H. (2009). InSTREAM: the individual-based stream trout research and environmental assessment model. *Gen. Tech. Rep. PSW-GTR-218. Albany, CA: US Department of Agriculture, Forest Service, Pacific Southwest Research Station. 254 p*, *218*.

Richter, A., & Kolmes, S. A. (2005). Maximum temperature limits for Chinook, Coho, and Chum Salmon, and steelhead trout in the Pacific Northwest. *Reviews in Fisheries Science, 13*(1), 23–49.

Sullivan, K., Martin, D. J., Cardwell, R. D., Toll, J. E., & Duke, S. (2000). An analysis of the effects of temperature on salmonids of the Pacific Northwest with implications for selecting temperature criteria. *Sustainable Ecosystems Institute, Portland, OR*.

von Biela, V. R., Bowen, L., McCormick, S. D., Carey, M. P., Donnelly, D. S., Waters, S., Regish, A. M., Laske, S. M., Brown, R. J., Larson, S., Zuray, S. & Zimmerman, C. E. (2020). Evidence of prevalent heat stress in Yukon River Chinook salmon. *Canadian Journal of Fisheries and Aquatic Sciences*, *77*(12), 1878-1892.

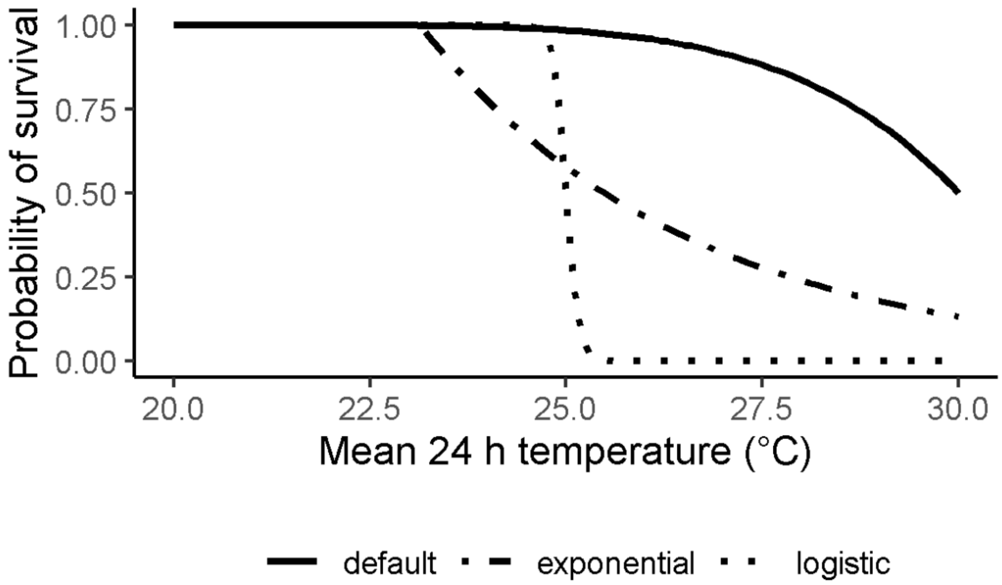

Figure S1. Acute temperature stress relationships between mean 24-h temperature and probability of survival. Default relationship based on the InSTREAM model (Railsback et al., 2009); exponential and logistic relationships modeled from equations and values in Jager (2011), and Sullivan et al. (2000).

Table S1. Percent of simulated fish experiencing mortality from acute temperature stress under the current and future thermalscape with and without cold-water refuges (CWRs) available (also see Figure S1). Default, exponential, and logistic are the temperature-mortality functions used in the uncertainty analysis (Jager, 2011; Railsback et al., 2009; Sullivan et al., 2000).

% Mortality	Default	Exponential	Logistic
Current, CWRs available	0.2	0.0	0.0
Current, CWRs unavailable	0.5	0.1	0.0
Future, CWRs available	1.1	18.9	0.0
Future, CWRs unavailable	1.9	28.0	0.0

Table S2. Percent of fish experiencing mortality and mean percent energy loss from for the secondary analysis where the probability of moving to cold-water refuge was increased based on relationship with the seasonal thermograph.

Condition Outcome	Default	Effect Strength A	Effect Strength B	Effect Strength C
% mortality	0.2	0.2	0.1	0.0
median % energy used	28	26	26	25
