# Supplementary material for: Tough places and safe spaces: Can refuges save salmon from a warming climate?
Source: Ecosphere. Author manuscript; Available in PMC 2023 Nov 9. (PMC9728623; doi:10.1002/ecs2.4265)
Supplement: Supplement2 [file NIHMS1850533-supplement-Supplement2.pdf]

Appendix S1. Depth and Temperature Data

Supplementary Information for Ecosphere: Tough places and safe spaces: can refuges save salmon from a warming climate?

Snyder, Marcía N.[1*], Schumaker, Nathan H.[1], Dunham, Jason B.[2], Ebersole, Joseph L.[1], Keefer, Matthew L.[3], Halama, Jonathan[1,4], Comeleo, Randy L.[1], Leinenbach, Peter[5], Brookes, Allen[1], Cope, Ben[5], Wu, Jennifer[5], Palmer, John[5]

[1]US Environmental Protection Agency, Pacific Ecological Systems Division, 200 SW 35th St., Corvallis, OR 97333

[2]US Geological Survey, Forest and Rangeland Ecosystem Science Center, 3200 SW Jefferson Way, Corvallis, OR 97331

[3]University of Idaho, Department of Fish and Wildlife Sciences, College of Natural Resources, 975 W. Sixth Street, Moscow, Idaho 83844

[4]Oak Ridge Institute for Science and Education/US Environmental Protection Agency, Pacific Ecological Systems Division, 200 SW 35th St., Corvallis, OR 97333

[5]US Environmental Protection Agency, Region 10, 1200 6th Ave., Suite 155, Seattle, WA 98101

*Corresponding author, ORCID: 0000-0003-2202-2668, email: snydermn@gmail.com, phone: 1-541-754-4423

Appendix S1. Depth and Temperature Data

Depth

Every riverine hexagon from Bonneville to McNary dam has an affiliated depth value. Several spatial datasets were used to assign depth values based on availability. For the mainstem Columbia River and the plume portion of each coldwater refuge, depth values were assigned from interpolated hydrographic survey data acquired from the U.S. Army Corps of Engineers Portland District. Tributary sections of coldwater refuges were based on interpolated field measurements of depth or attributed depth values based on linear relationships between volume and mean channel width.

Temperature

Here we describe our approach for compiling continuous hourly temperature time series for the Columbia River and important coldwater refuge tributaries. The model uses hourly water temperatures from July 1 - October 31. For calibration model runs, we simulated a time series of "typical, recent" conditions for the Columbia River, tributaries, and tributary plumes. For model applications we based the current (present-day) and future temperature time series for the mainstem Columbia River on a specific year (2017) which was relatively warm to better capture daily temperature minima and maxima.

Calibration Temperature Time Series

Available hourly temperature data for coldwater refuge tributaries to the Columbia River and from the Columbia River data were acquired from the NorWeST project database (https://www.fs.fed.us/rm/boise/AWAE/projects/NorWeST/StreamTemperatureDataSummaries.

shtml). The temperature data had previously been assessed for quality by NorWeST project staff. Additional sources of data included temperature readings from EPA R10 field crews in 2016, and temperatures recorded by other entities but not incorporated in the NorWeST dataset. Temperature data includes sites monitored from 1991 to 2016. Data were consolidated by location, year, date, and hour.

Here we describe our method for estimating temperatures at locations without monitoring data and for interpolating missing data. Hourly temperature data were recorded by federal, state and local governments, non-profits, and others from streams within the study area, and the duration and timing of temperature monitored was not uniform across sites. As a result, many temperature measurements for a given reach have voids in data coverage. Additionally, many coldwater tributary plumes or sections of tributary confluence areas lack temperature data.

Columbia River temperatures

The spatial extent of the model is from the Bonneville Reservoir to the Snake River confluence. Each reservoir and the section between McNary dam and the confluence are represented in the model as having the same temperature throughout because they are well-mixed and without much temperature variability between upstream and downstream points. To characterize temperature of each of the four mainstem Columbia River sections, we averaged temperatures for each hour on a given date and across years between July 1 and October 31 for the ten available temperature data collection sites from the NorWest dataset.

Tributary temperatures

Because measured temperatures were not available on every date for every year for all sites, to characterize typical temperature for a given hour on a given date for each individual tributary stream, we averaged measured temperatures for each hour across all years between July 1 and October 31 for each site. This resulted in a value that is a mean of the hourly temperature, for each day, across all years for which that tributary was monitored. Therefore, temperatures represent 'typical' temperatures for that tributary over the period of measurement and not specific temperatures for any particular year.

Some sites are missing data for hour/dates between July and October. To interpolate missing data, we estimated a mean hourly temperature for all tributary locations combined for which continuous hourly water temperatures were available for the July – October time period. We calculated the ratio between the calculated mean (of complete time series) and the observed temperature of individual incomplete time series for the period of temporal overlap. We estimated missing hourly temperature for incomplete individual tributaries by multiplying the mean ratio by the mean hourly temperature for the period of missing data (following Butcher et al. 2010).

Plume Temperatures

We estimated temperatures within the plumes of tributaries at their confluence with the Columbia River and within coves as a linear function of hourly tributary temperatures and hourly Columbia River temperatures. Coves were plumes in natural or human made embayments that partially protected plumes from immediate mixing with the Columbia River. The mixing of tributary and Columbia River flows as well as localized heating of tributary water in the confluence zone determine the temperature of the plume and cove regions. We presupposed that

temperatures within plumes and coves will be intermediate between the temperature of the tributary and the temperature of the Columbia. While plumes are spatially heterogeneous in temperature, we necessarily simplified plume temperatures to a single temperature within the plume region for any model hour and day of the year. The objective of this interpolation was to estimate an average plume temperature that represents the mean temperature that could be experienced by fish occupying some region of the plume between its edge at the Columbia and the tributary itself.

We defined the plume spatial extent using field measurements, modelling with CORMIX software (http://www.cormix.info/), or via extrapolating from modeled plume relationships. Coldwater refuges were defined as regions 2°C colder than the Columbia River based on mean August water temperature. We determined plume volumes using this definition meaning that plumes were drawn out from each tributary to a point where the plume warmed to within 2°C of the mean August water temperature of the Columbia River ($T_{Columbia}$). For each plume modeled with CORMIX and those modeled by extrapolation of CORMIX, results contain a temperature associated with the tributary (T_{trib}) and the modeled plume edge (T_{edge}), with a gradient of temperatures between the two edges. The temperature of the plume edge (T_{edge}) is defined as a function of the Columbia River temperature ($T_{Columbia}$) and the tributary temperature (T_{trib}) multiplied by a mixing factor, *b*.

$$T_{edge} = T_{Columbia} - [b*(T_{Columbia} - T_{tributary})]$$ **Eq. S1**

Using the mean August temperatures defined for each plume we then solved for *b*. We calculated plume edge temperatures as a function of tributary and Columbia River temperature for all paired hourly observations while assuming *b* is constant over time. We took the mean of the plume edge and tributary temperatures to estimate spatially-averaged hourly temperatures within plume and

cover areas (T_{plume}) by assuming a linear gradient of temperature between the tributary and plume edge:

$$T_{plume} = (T_{edge} - T_{tributary})/2 \quad \textbf{Eq. S2}$$

We used this as a scaling factor to capture the relative effect of each tributary on its associated plume temperature.

Model Application Time Series

Columbia River temperatures

For the current and future model application time series, we considered each reservoir and the section between McNary dam and the confluence as having the same temperature throughout because they are well-mixed and without much temperature variability between upstream and downstream points. Rather than collating interpolated hourly data from NorWest, we used ACOE DART measured temperature observations from the hydropower dam tailrace and forebays (http://www.cbr.washington.edu/dart) to represent the four mainstem Columbia River locations. The current thermalscape model simulations were based on measured temperature from the year 2017. Future thermalscape model simulations represent 2040 predicted temperature increases at (0.5 °C per decade) and so added 1°C to the current hourly time series. The Bonneville reservoir temperature time series was estimated with hourly DART data for Dalles tailrace. The Dalles reservoir temperature time series was estimated with hourly DART data for John Day tailrace. The John Day reservoir temperature time series is created using hourly DART data for the John Day forebay. Upstream of McNary dam to the Snake River confluence is based on McNary tailrace temperature.

Tributary and plume temperature

For model application simulations, future and current, tributary and plume temperatures were estimated based on NorWest measured temperatures. The same temperatures were used for the tributaries and plumes for the model calibration and model application simulations.

Assumptions and limitations

The estimation approach does not account for spatial variation within plumes. Differential heating, as well as currents and differential movement of tributary flows within the plume region will result in heterogeneous temperatures that are necessarily simplified in this approach. More sophisticated modeling approaches informed by spatially and temporally intensive monitoring would be needed to better approximate plume temperatures at finer scales. Our approach assumes a constant mixing ratio between the tributary and the Columbia, based upon mean August conditions. The ratio likely varies depending on relative discharge between the tributary and Columbia and other factors that we are unable to account for at this time.

Reference

Butcher, D., Crown, J., Brannan, K., Kishida, K. and Hubler, S. 2010. John Day River basin total maximum daily load (TMDL) and water quality management plan (WQMP). Oregon Department of Environmental Quality, Portland, Oregon, USA.
