# Supplementary material for: Tough places and safe spaces: Can refuges save salmon from a warming climate?
Source: Ecosphere. Author manuscript; Available in PMC 2023 Nov 9. (PMC9728623; doi:10.1002/ecs2.4265)
Supplement: Supplement3 [file NIHMS1850533-supplement-Supplement3.pdf]

Appendix S2. Dam Passage

Supplementary Information for Ecosphere: Tough places and safe spaces: Can refuges save salmon from a warming climate?

Snyder, Marcía N.[1*], Schumaker, Nathan H.[1], Dunham, Jason B.[2], Ebersole, Joseph L.[1], Keefer, Matthew L.[3], Halama, Jonathan[1,4], Comeleo, Randy L.[1], Leinenbach, Peter[5], Brookes, Allen[1], Cope, Ben[5], Wu, Jennifer[5], Palmer, John[5]

[1]US Environmental Protection Agency, Pacific Ecological Systems Division, 200 SW 35th St., Corvallis, OR 97333

[2]US Geological Survey, Forest and Rangeland Ecosystem Science Center, 3200 SW Jefferson Way, Corvallis, OR 97331

[3]University of Idaho, Department of Fish and Wildlife Sciences, College of Natural Resources, 975 W. Sixth Street, Moscow, Idaho 83844

[4]Oak Ridge Institute for Science and Education/US Environmental Protection Agency, Pacific Ecological Systems Division, 200 SW 35th St., Corvallis, OR 97333

[5]US Environmental Protection Agency, Region 10, 1200 6th Ave., Suite 155, Seattle, WA 98101

*Corresponding author, ORCID: 0000-0003-2202-2668, email: snydermn@gmail.com, phone: 1-541-754-4423

Appendix S2. Dam Passage

Parameters

In the model, simulated fish moving through hydropower dams pass through tailrace and fish ladder areas. Fish swim speed and energy use is modified by tailrace and fish ladder model regions. Simulated fish pass through the Dalles, John Day, and McNary tailrace and fish ladders. In the model, energy use and passage time modifications are equal for all dams and populations. Dam passage does not include fall back. During passage through the tailrace and fish ladder energy use is increased by factors of 1.62, 1.26, respectively based on data in Keefer et al. (2017). Each individual's swim speed is modified for the tailrace and fish ladder to reflect observed passage times. Tailrace passage times used in the simulation are based on tagged fish data summarized in Crozier et al. (2017). Modeled passage time distributions were assigned based on arrival timing at the tailrace, which were simplified from the six arrival time periods from Crozier et al. (2017) to 'day' or 'night'. Each arrival time was then subdivided into short, average, and long duration time distributions based on the estimated proportion of fish at each arrival time exhibiting that passage time which was also estimated from Crozier et al. (2017) (Table S1). For fish arriving during 'day' time model hours 0:00-17:00, 40% short, 40% average, and 20% long duration were assigned. For fish arriving during the 'night', model hours 17:00-24:00, 20% short, 60% average, and 20% long duration were assigned. Hourly mean, standard deviation, minimum, and maximum values for fish arriving during the 'day' were based on observed fish arriving from 8:00-12:00 and 'night' arrivals were based on observed fish arriving from 16:00-20:00 (Crozier et al. 2017).

Table S1. Tailrace passage time distributions were assigned based on arrival timing at the tail race and subdivided into short, average, and long durations.

Arrival Timing	Duration	Mean	Standard Deviation	Minimum	Maximum
day	Short	6	2.7		
day	Average	21.6	4.5		
day	Long			30	76.8
night	Short	3.6	1.2		
night	Average	15.6	5.4		
night	Long			27.8	76.8

Dam Passage Tuning

The primary emergent parameter we evaluated for calibration of dam structures was total passage time through the combined dam structures. Passage time through dam structures includes time spent in the tailrace and fish ladder portion of each hydropower structure. Model calibration performed with temperature values from the current scenario were meant to represent recent temperature values for the Columbia, cold water refuges plumes and tributaries. Temperature values were modeled as mean hourly values and include data from 1992-2016 (see Appendix S1 for details). We iteratively adjusted the mean passage time through the fish ladder. The range of values for mean fish ladder passage time are based on Keefer et al. (2017). Observed median dam passage time (19 h) was summarized in Keefer et al. (2017). Modeled

median dam passage time after calibration (21 h) is within 17% of the observed passage time (Table S2).

Table S2. Comparison of simulated total dam passage time to observed dam passage time from (Keefer et al. 2017). Percent difference was calculated as the modeled value minus observed divided by the observed value.

**Percentile**	**Observed Total Dam Passage Time (h)**	**Modeled Total Dam Passage Time (h)**	**% Difference**
5%	6	5	17
25%	10	11	10
50%	19	21	11
75%	29	32	10
90%	85	71	16

Literature Cited

Crozier, L. G., Bowerman, T. E., Burke, B. J., Keefer, M. L., & Caudill, C. C. (2017). High-stakes steeplechase: a behavior-based model to predict individual travel times through diverse migration segments. *Ecosphere, 8*(10), p. e01965.

Keefer, M.L., Jepson, M.A., Clobough, T.S., Caudill, C.C., Bjornn, T.C., Burke, B.J., Matter, A.L., Moser, M.L., Perry, C.A. and Stuehrenberg, L.C. (2017). Migration of adult

salmonids in the federal Columbia River hydrosystem: a summary of radiotelemetry studies, 1996-2014 (No. 2017-2). US ACOE Portland District.
